# Enhancing vehicle performance through the application of airfoils as spoilers with movable trailing edge

**DOI:** 10.12688/f1000research.160307.1

**Published:** 2025-04-28

**Authors:** Ahmad Karaki, Mohammad Abu Sirreya, Majdi Zalloum, Husein Amro

**Affiliations:** 1 mechanical engineering, Palestine Polytechnic University, Hebron, Palestinian Territory

**Keywords:** CFD, Simulation, Vehicle Dynamics, Spoiler, Trailing Edge.

## Abstract

**Background:**

Vehicle safety and stability are paramount in the automotive industry, with aerodynamics playing a crucial role in enhancing these attributes. Spoilers, often used to modify airflow around vehicles, can significantly impact performance depending on their design and configuration. This study explores the use of airfoils as spoilers with adjustable trailing edges to dynamically control downforce and lift forces, aiming to improve vehicle stability and performance.

**Methods:**

Computational Fluid Dynamics (CFD) simulations were performed using ANSYS Fluent ® software (ANSYS, Inc., Canonsburg, PA, USA) The authors confirm that they have obtained the necessary license for the use of this software in academic research. to analyses the aerodynamic effects of spoilers with varying trailing edge angles (AOTE). A Tesla car model was designed in CATIA™ software (Dassault Systèmes), Vélizy-Villacoublay, France), and simulations were conducted at speeds ranging from 120 km/h to 350 km/h. The Shear Stress Transport (SST) k-ω turbulence model was employed to accurately capture airflow patterns. The computational domain was configured as a wind tunnel, and a grid independence study ensured the reliability of the results. Boundary conditions included velocity inlets, pressure outlets, and no-slip walls for the car and spoiler surfaces.

**Results:**

The study revealed that adjusting the trailing edge angle had a significant impact on downforce and lift forces. At a trailing edge angle of 30 degrees, the negative lift force increased by up to 36%, while at zero degrees, it increased by up to 17%. The positive lift force was optimized to enhance vehicle performance during acceleration, resulting in an overall increase in total lift force by up to 15%. The simulated drag coefficient of 0.256 showed a 6% discrepancy compared to Tesla’s reported value of 0.24, primarily due to differences in mesh refinement and the omission of certain design features.

**Conclusions:**

This study demonstrates the potential of movable trailing edge spoilers in improving vehicle stability, handling, and acceleration. The ability to dynamically adjust aerodynamic forces offers a practical solution for enhancing vehicle performance and safety. Future research will focus on refining control algorithms and testing under a broader range of driving conditions. These findings provide a strong foundation for integrating advanced aerodynamic features into modern vehicle designs.

## Introduction

Automotive spoilers, once primarily associated with high-performance sports cars and racing vehicles, have become a common sight on a wide range of automobiles. These devices, typically mounted on the rear of a car, have gained popularity among car enthusiasts for their purported ability to enhance vehicle performance, stability, and fuel efficiency. This study delves into the effect of spoilers on car performance to provide a comprehensive understanding of their impact.

Spoilers, as aerodynamic appendages, are designed to modify the airflow around a vehicle. They can take various forms, including lip spoilers, wing spoilers, and even roof spoilers, each designed with distinct aerodynamic objectives. Some enthusiasts believe that the addition of a spoiler to a car can improve its speed, cornering capabilities, and fuel economy. However, the efficacy of these modifications depends on factors such as the design, size, and installation of the spoiler, as well as the specific characteristics of the vehicle it is attached to.

The primary goal of this study is to systematically investigate the influence of spoilers on various aspects of car performance, shedding light on the practical implications of their use. The investigation covers aerodynamic effects, handling, and stability, with the aim of providing empirical evidence to support or refute the claims associated with spoilers.

As automotive technology continues to advance, and with a growing focus on energy efficiency and environmental impact, understanding the role of spoilers in vehicle performance is crucial. This study contributes to the body of knowledge in the automotive field and helps dispel myths surrounding the effects of spoilers on cars.

In the subsequent sections of this study, delve into the research methodology, data collection, and analysis, with the aim of providing a comprehensive and evidence-based evaluation of the effect of spoilers on vehicle performance. The results of this investigation have the potential to influence future automotive design considerations, consumer choices, and the broader understanding of the role of spoilers in the automotive industry.

This study embarks on a comprehensive exploration of the effects of spoilers on vehicle performance, using a combination of CATIA for design and ANSYS for computational fluid dynamics (CFD) simulations to provide a holistic understanding.

## Literature review

Once exclusive to high-performance sports cars, spoilers have become common on various vehicles, sparking curiosity about their impact beyond aesthetics. Researchers have delved into the intricacies that define how spoilers influence modern vehicles.

In aerodynamics, studies by Smith and Johnson ^1^ carefully examined spoiler designs, decoding their effects on airflow and downforce. This research goes beyond aesthetics, aiming to understand how spoilers enhance a vehicle’s aerodynamic efficiency.

Patel and Garcia ^2^ further explored this by using Computational Fluid Dynamics (CFD) simulations to scrutinize fluid dynamics and pressure associated with spoilers. Their work provides insights into optimizing spoiler aerodynamics.

Simultaneously, Thompson et al. ^3^ empirically explored the real-world impact of spoilers on speed and handling. Their studies quantified how different spoiler configurations influence acceleration, braking, and cornering forces, providing tangible data on dynamic behaviour.

Chen et al. ^4^ investigated vehicle stability and rollover prevention, acknowledging that spoilers contribute to safety. They explored parameters such as steering input, speed, and ground friction to design spoilers that enhance stability and reduce rollover risks.

In tandem, other scholars broadened the scope. Green et al. ^5^ assessed the ecological footprint of spoiler design, considering energy efficiency and sustainability. Concurrently, studies on driver preferences ^6^ balanced aesthetics and functional efficacy in spoiler design.

As the automotive industry continues to advance, tools like MATLAB and ANSYS have been employed to unravel the interplay between spoilers and control mechanisms. This integration optimizes not only aerodynamic performance but also overall efficiency and safety.

While existing literature provides valuable insights, this study offers a detailed methodology. By presenting an evidence-based evaluation, researchers aim to contribute to and redefine how spoilers, with evolving designs and technologies, shape the performance landscape of contemporary automobiles.

## Airfoil

Airfoil is a shape designed to generate lift when air flows over it. The shape is typically found in cross-sections of wings, blades, and other surfaces that move through air, such as aircraft wings, helicopter rotor blades, propellers, and wind turbines.

**Figure 1.**
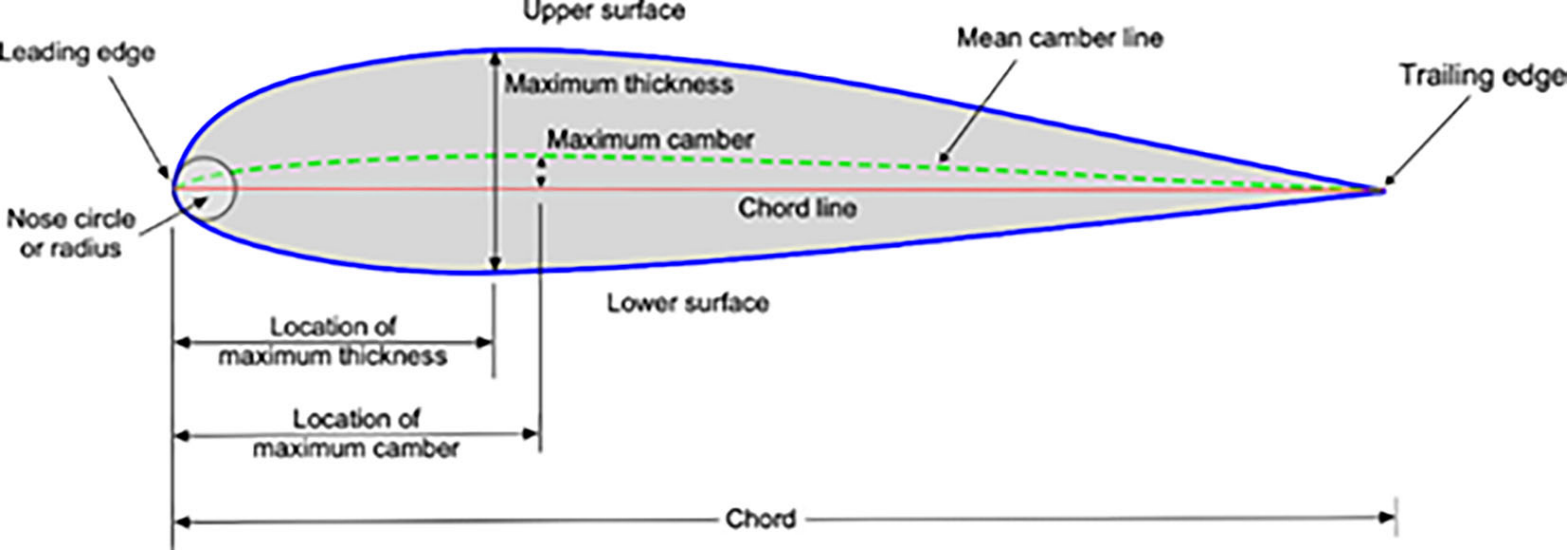
Airfoil geometry.

Figure 1 presents graphical depictions of key aerodynamic features associated with an airfoil, offering insights into its geometry. The following elements are typically illustrated:

(1) **Camber line**:
  The camber line represents the curve defining the maximum distance between the upper and lower surfaces of the airfoil. It characterizes the camber or curvature of the airfoil. This line is crucial in determining the lift characteristics of the airfoil, as it influences the distribution of pressure around the surface.
(2) **Chord line**:
  The chord line is a straight line connecting the leading edge to the trailing edge of the airfoil. It serves as a fundamental reference for aerodynamic analysis.
  The angle of attack, a parameter crucial to lift generation, is typically measured with respect to the chord line.
(3) **Thickness**:
  The thickness of the airfoil is the maximum distance between the upper and lower surfaces, perpendicular to the chord line. This dimension contributes to the overall aerodynamic characteristics and structural considerations of the airfoil.
(4) **Trailing edge**:
  The rear edge of an airfoil is where the airflow separated by the Leading Edge regions and where the essential control surfaces are located.

### Airfoil group

Understanding the aerodynamic characteristics of different airfoil shapes is crucial for the design and performance optimization of aircraft wings. Airfoils are the cross-sectional shapes of wings and are designed to produce lift efficiently while minimizing drag. Table 1 presents a comprehensive comparison of various airfoil groups, detailing their geometric and aerodynamic properties.

**Table 1.** Airfoil group tables and analyses.

Group A					
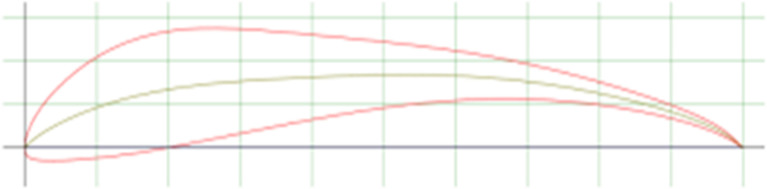					
Name	Thick	Camber	Chord	CD	CL
**S1223RTL**	13.5	8.3	0.3 m	0.047	1.019
**GOE243**	14.2	9.2	0.3 m	0.052	1.135
**FX73-CL3-152**	15.2	8	0.3 m	0.057	0.985
**USA32AIRFOIL**	14.7	9.3	0.3 m	0.068	1.146

Group B					
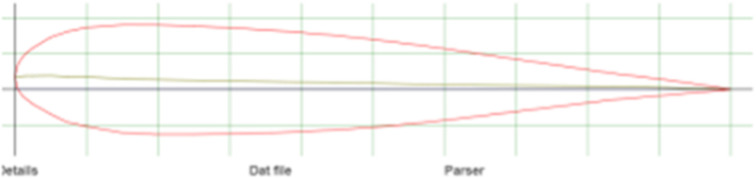					
Name	Thick	Camber	Chord	CD	CL
**NREL5801**	13.5	3.7	0.3 m	0.027	0.457
**EPPIER434**	13.3	4.3	0.3 m	0.028	0.531
**AH63-IC-127\24**	12.7	3.8	0.3 m	0.025	0.47
**AH93-K-130\15**	13.1	3.5	0.3 m	0.025	0.433

Group C					
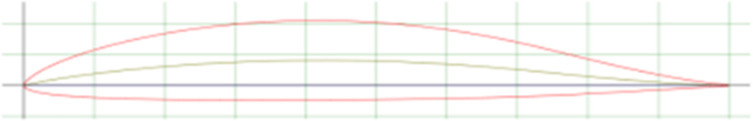					
Name	Thick	Camber	Chord	CD	CL
**DoEing737 root**	15.4	0.2	0.3 m	0.027	0.024
**OAF139**	13.7	0	0.3 m	0.024	0
**-E474**	14.1	0	0.3 m	0.025	0
**-EPPIER479**	16.6	0	0.3 m	0.028	0

Group D					
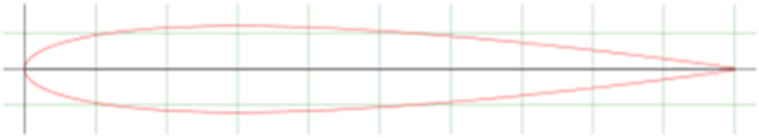					
Name	Thick	Camber	Chord	CD	CL
**NACA0012**	12	0	0.3 m	0.019	0
**-EPPLER EA(6)**	12	0	0.3 m	0.019	0
**-RAF30**	12.6	0	0.3 m	0.021	0
**-GOE 409**	12.7	0	0.3 m	0.021	0

This data was taken: at a speed of 120 km/h, altitude = 0 and wing area = 0.3 m ^2^.

As shown in Table 1, the NACA0012 airfoil was selected for its symmetrical properties. This design rationale hinges on adopting a spoiler configuration distinguished by symmetrical geometric attributes. Within this framework, the lift force demonstrates asymptotic convergence towards nullity, displaying minimal sensitivity to variations in airspeed. The inherent geometric symmetry of the spoiler facilitates meticulous control over lift force modulation during spoiler articulation, ensuring precision in both lift augmentation and attenuation.

This particular design subtlety assumes significance owing to its contribution to heightened accuracy in lift force management, regardless of the directional adjustments (upward or downward). The deliberately engineered symmetrical spoiler configuration confers stability and predictability to the nuanced control of lift forces, providing a refined and controlled response across diverse aerodynamic conditions. This precision proves integral to optimizing the spoiler’s performance, especially during manoeuvres that demand exacting lift adjustments.

### CATIA model

In this section, the shapes provide an illustration of the shape of the spoiler with different angles of trailing edge.

**Figure 2.**
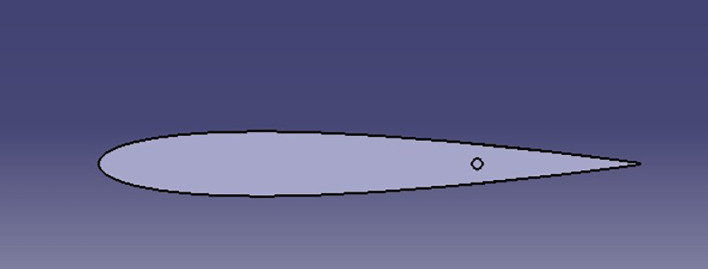
Spoiler model at 0 angle of trailing edge.

**Figure 3.**
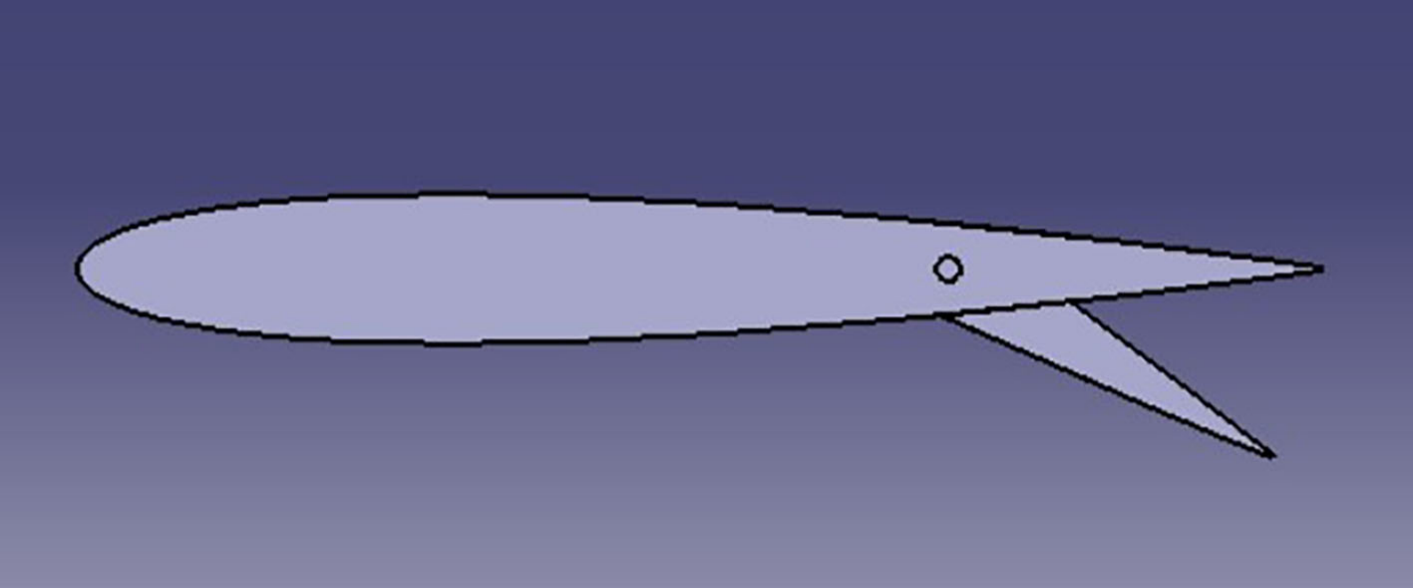
Spoiler CATIA model at negative angle of trailing edge.

**Figure 4.**
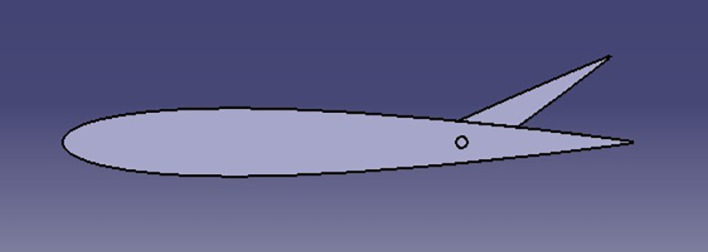
Spoiler CATIA model at positive angle of trailing edge.

## Computational Fluid Dynamics (CFD)

It is a branch of fluid mechanics that uses numerical methods and algorithms to solve and analyse problems involving fluid flow. Fluid flow can occur in various contexts, such as air flow around an aircraft, water flow in rivers, heat transfer in pipes, and many others.

CFD simulations involve dividing the fluid domain into a grid or mesh of small elements and solving mathematical equations that govern fluid flow, heat transfer, and other related phenomena. These equations, which describe the conservation of mass, momentum, and energy, are solved iteratively to obtain a numerical solution that represents the behaviour of the fluid.

In the automotive context, CFD is utilized to simulate and analyse the airflow around a car’s body and various components. Engineers can assess the impact of different design elements, such as spoilers, body shapes, and side mirrors, on aerodynamic performance. By virtually testing these components through CFD simulations, engineers can optimize designs to achieve goals like minimizing drag, maximizing downforce, and enhancing overall aerodynamic efficiency.

In summary, the integration of CFD in the automotive industry, particularly in the context of cars, allows engineers to optimize aerodynamic performance by virtually testing and refining designs. This approach significantly reduces the need for expensive and time-consuming physical experiments, leading to more efficient and effective vehicle designs.

### Turbulence model ( *SST k*- *ω Model*)

The Shear Stress Transport (SST) *k*- *ω* turbulence model, introduced by Menter, ^7^ is a widely used two-equation eddy-viscosity model known for its versatility in simulating complex flows. This model effectively combines the strengths of the *k*- *ω* and *k*- *ε* formulations. In the near-wall region, the *k*- *ω* formulation is employed, allowing the model to accurately capture boundary layer effects without requiring additional damping functions. In the free-stream region, the model transitions to *k*- *ε* behaviour, addressing the sensitivity issues often associated with traditional *k*- *ω* models when dealing with free-stream turbulence properties.

One of the key advantages of the SST k-ω model is its robust performance in flows with adverse pressure gradients and separation, making it particularly suitable for simulating the complex airflow patterns around vehicle spoilers. However, the model tends to overpredict turbulence levels in regions with high normal strain, such as stagnation points or areas of strong acceleration. Despite this limitation, the SST k-ω model remains a preferred choice for aerodynamic simulations due to its overall accuracy and reliability. ^8^

**Figure 5.**
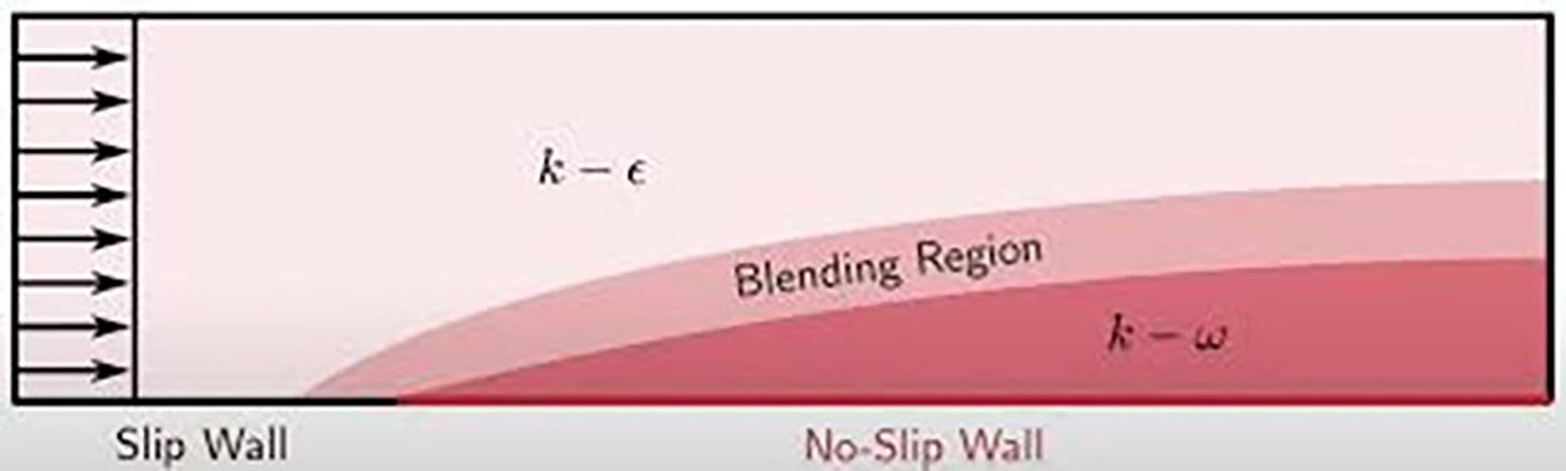
Turbulence model.

Kinematic Eddy Viscosity:

$$v_{T}=\frac{a_{1}k}{\max\left(a_{1}\omega ,SF_{2}\right)}$$ (1)

Where:

*a*_1_: Model constant, typically around 0.31.

***S***: Strain rate magnitude.

Turbulence Kinetic Energy:

$$\frac{\mathrm{\partial k}}{\mathrm{\partial t}}+U_{j}\frac{\mathrm{\partial k}}{\partial x_{j}}=P_{k}-\beta^{*}\mathrm{k\omega}+\frac{\partial }{\partial x_{j}}\left[\left(v+\sigma_{k}v_{T}\right)\frac{\mathrm{\partial k}}{\partial x_{j}}\right]$$ (2)

Specific Dissipation Rate:

$$\frac{\partial \omega }{\mathrm{\partial t}}+U_{j}\frac{\partial \omega }{\partial x_{j}}=\alpha S^{2}-\beta \omega^{2}+\frac{\partial }{\partial x_{j}}\left[\left(v+\sigma_{\omega }v_{T}\right)\frac{\partial \omega }{\partial x_{j}}\right]+2\left(1-F_{1}\right)\sigma_{\omega 2}\frac{1}{\omega }\frac{\mathrm{\partial k}}{\partial x_{j}}\frac{\partial \omega }{\partial x_{j}}$$ (3)

Where:

*k*: Represents the energy contained in turbulent eddies.

*P*: Production of *k*, usually due to velocity gradients.

*β* ^∗^: Model constant, controls the dissipation rate of *k* to *ω.*

*ω*: Specific dissipation rate.

*v*: Kinematic viscosity.

*σ*: Model constant, controls the turbulent diffusion of *k.*

*v* *_t_*: Turbulent viscosity.

Closure Coefficients and Auxiliary Relations:

$$F_{2}=\tanh\left[{\left[\max\left(\frac{2\sqrt{k}}{\beta^{*}\mathrm{\omega y}},\frac{500v}{y^{2}\omega }\right)\right]}^{2}\right]$$ (4)

$$P_{K}=\min\left(\tau_{\mathrm{ij}}\frac{\partial U_{i}}{\partial x_{i}},10\beta^{*}\mathrm{k\omega}\right)$$ (5)

$$F_{1}=\tanh\left\{{\left\{\min\left[\max\left(\frac{2\sqrt{k}}{\beta^{*}\mathrm{\omega y}},\frac{500v}{y^{2}\omega }\right),\frac{4\sigma_{\omega 2}k}{{\mathrm{CD}}_{\mathrm{k\omega}}y^{2}}\right]\right\}}^{4}\right\}$$ (6)

$${\mathrm{CD}}_{\mathrm{k\omega}}=\max\left(2\rho \sigma_{\omega 2}\frac{1}{\omega }\frac{\mathrm{\partial k}}{\partial x_{i}}\frac{\partial \omega }{\partial x_{i}},10^{-10}\right)$$ (7)

$$\alpha_{1}=\frac{5}{9},\alpha_{2}=0.44$$ (8)

$$\beta_{1}=\frac{3}{40},\beta_{2}=0.44$$ (9)

$$\beta^{*}=\frac{9}{100}$$ (10)

$$\sigma_{k1}=0.85,\sigma_{k2}=1$$ (11)

$$\sigma_{\omega 1}=0.5,\sigma_{\omega 2}=0.856$$ (12)

• **Near-Wall Treatment**: In the near-wall region, *F*1≈1 ensures that the *k*- *ω* model is used, which is more accurate for capturing the effects close to the wall.
• **Away from the Wall**: Further away from the wall, F1 decreases towards 0, blending towards the *k*- *ϵ* model, which is better suited for free-stream turbulence.
• **Turbulent Viscosity Adjustment**: *F*2 further refines the turbulent viscosity calculation to ensure smooth and accurate transitions between the near-wall and far-field regions.

## Ansys simulation and result analysis

This chapter presents the results of aerodynamic simulations conducted using ANSYS Fluent ^®^ software (ANSYS, Inc., Canonsburg, PA, USA). Two primary configurations were analyzed: a baseline car model without any modifications and a modified car model equipped with a spoiler featuring adjustable trailing edge angles (AOTE). The simulations were performed to evaluate the impact of spoiler adjustments on vehicle aerodynamics and stability.

The car model was designed using CATIA, and the computational domain was created to represent a wind tunnel. The simulations were conducted at various speeds, ranging from 120 km/h to 350 km/h, to assess the aerodynamic forces under different driving conditions. The Shear Stress Transport (SST) *k*- *ω* turbulence model was employed to capture the complex airflow patterns around the vehicle and spoiler.

### Car model

**Figure 6.**
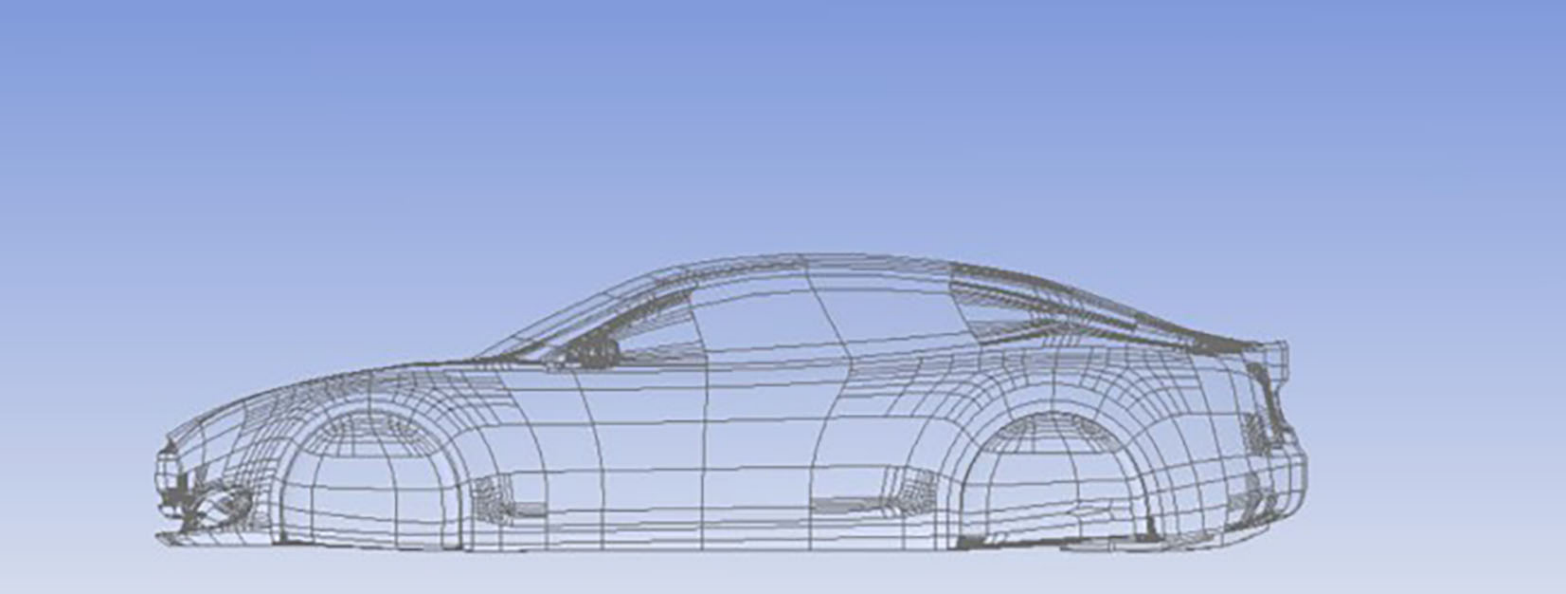
Car model.

A simple Tesla model was drawn on CATIA software, which is shown in Figure 6.

### Car model with spoiler

The design of the spoiler type is NACA0012, 120*45 cm with different AOTE.

**Figure 7.**
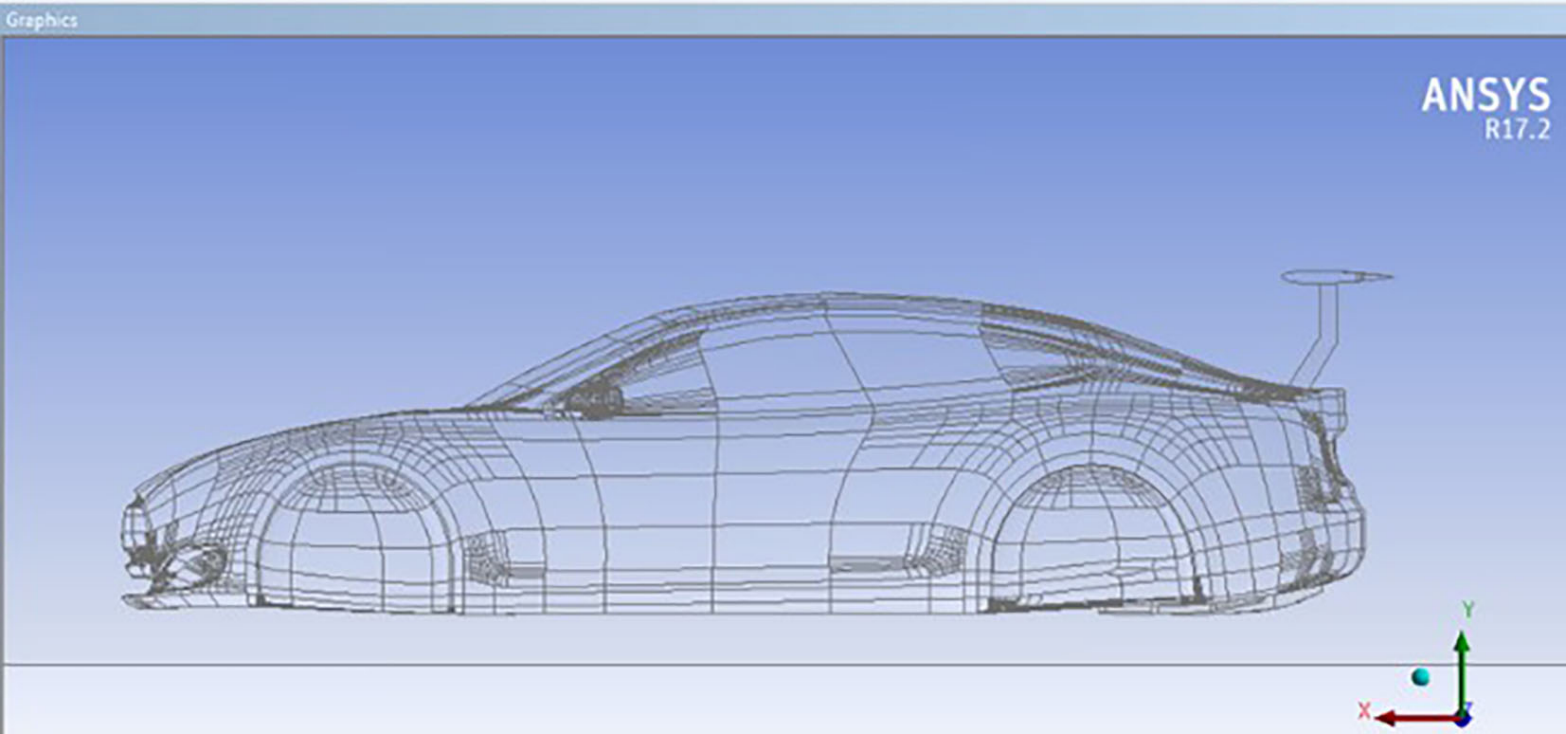
Car model with spoiler.

### Process procedure

**Figure 8.**
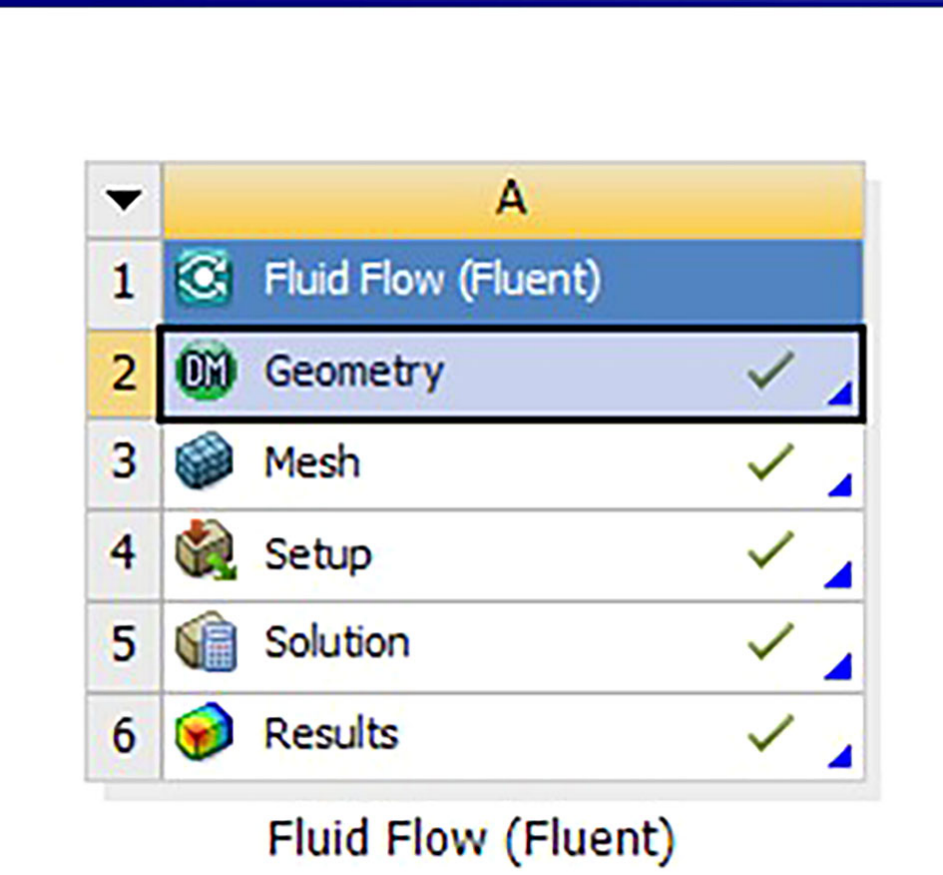
Sequence of processes in ANSYS.

The ANSYS simulation process involves several sequential steps to ensure accurate and reliable results. These steps include pre-processing, meshing, setting up boundary conditions, and selecting the appropriate turbulence model. Each stage is critical for achieving a realistic representation of the aerodynamic behaviour of the vehicle. The sequence of processes in the ANSYS Workbench is illustrated in Figure 8.

**Figure 9.**
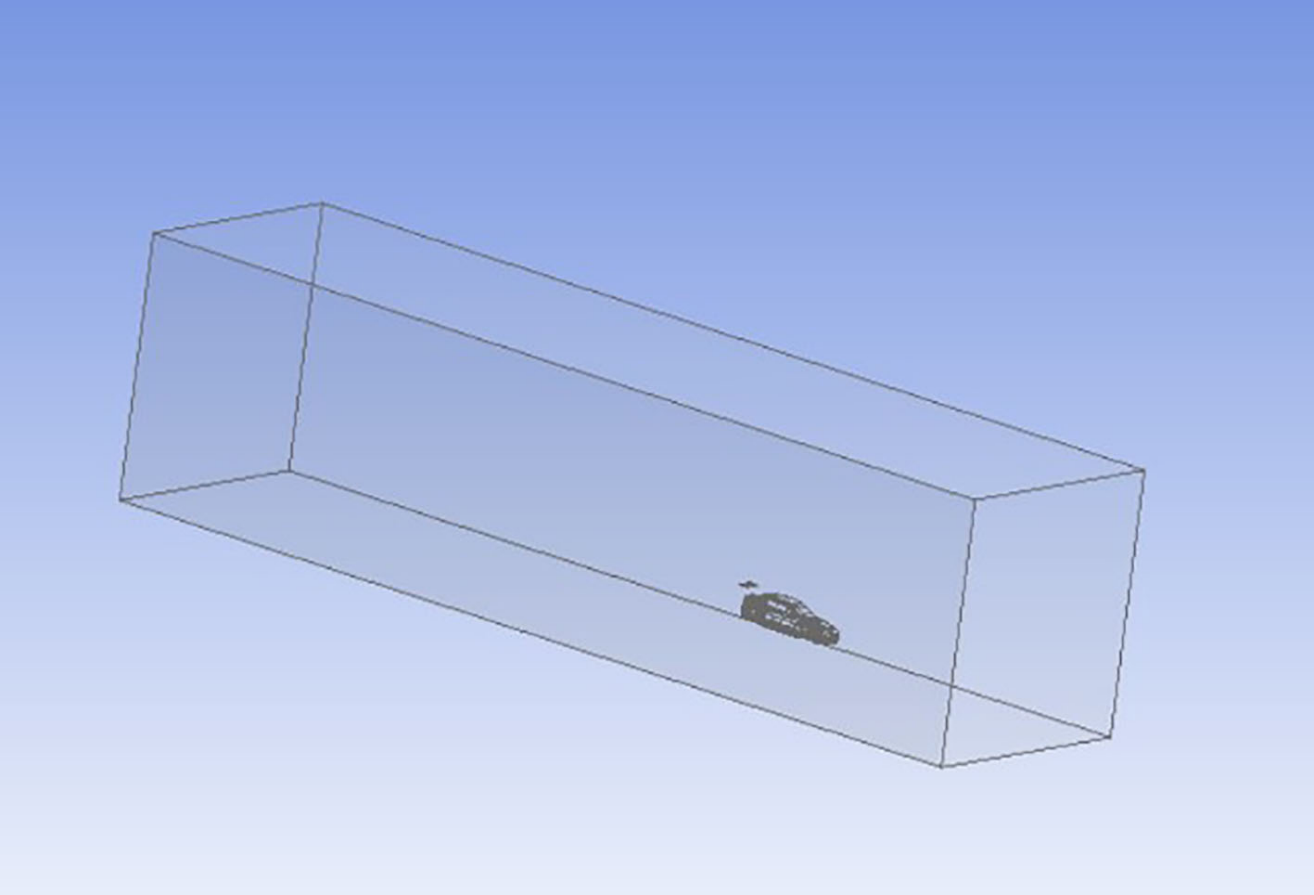
Enclosure.

**Pre-processing **:

The first step in the simulation process is pre-processing, where the car model is prepared for analysis. The 3D model of the car, designed in CATIA, is imported into ANSYS Geometry. A computational domain, or enclosure, is then created around the car to simulate a wind tunnel environment. The dimensions of the enclosure are provided in Table 2, and the enclosure is visualized in Figure 9.

**Table 2.** Enclosure geometry.

X	Y	Z
15 m	10 m	10 m
-25 m	-0.2 m	-10 m

**Figure 10.**
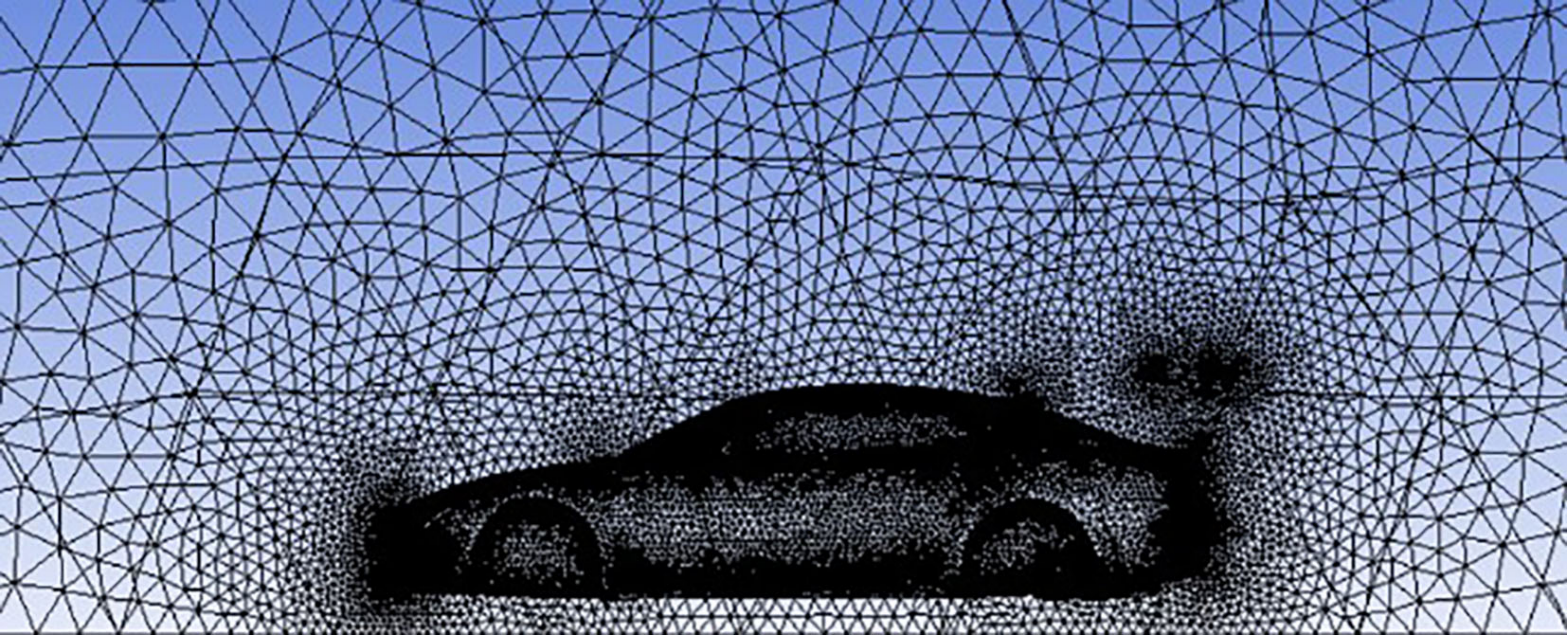
Car model after meshing process.

**Meshing**:

The next step is meshing, where the computational domain is divided into a grid of small elements. Proper meshing is essential for accurate results, as an improperly meshed model can lead to incorrect solutions. Special attention is given to refining the mesh around the car and spoiler, where the interaction between air molecules and the vehicle is most significant. The meshed model is shown in Figure 10.

**Boundary conditions**:

After meshing, the boundary conditions are defined to simulate real-world airflow conditions. The inlet is set as a velocity inlet with speeds ranging from 120 km/h to 350 km/h, while the outlet is defined as a pressure outlet. No-slip boundary conditions are applied to the car and spoiler surfaces, and symmetry conditions are used to reduce computational cost. The details of the boundary conditions are provided in Table 3.

**Table 3.** Enclosure.

name	Boundary type	Boundary details
Inlet	Velocity-inlet	Normal speed, fore cases: 120 km/h, 180 km/h, 250 km/h, 300 km/h and 350 km/h. turbulence: medium (intensity = 5%).
Outlet	Pressure-outlet	Absolute
Wall	Wall	No slip
Symmetry	Symmetry	Half of enlister
Car	Wall	No slip

**Turbulence model**:

The final step in the setup process is selecting the turbulence model. The Shear Stress Transport (SST) *k*- *ω* model is chosen for its ability to accurately capture the complex airflow patterns around the vehicle, particularly in regions with adverse pressure gradients and flow separation.

### Result analysis and discussion

After finishing the fourth pre-process step, the model was ready to do the final step, which is the solution to get the results.

**Streamline and pressure contour for car model and spoiler with difference AOTE:**

The following figure illustrates the airflow streamlines and pressure contour around a car model equipped with a spoiler at various angles of trailing edge (AOTE). As the AOTE changes, the behaviour of the airflow around the car and spoiler also changes, influencing the aerodynamic forces such as lift, drag, and downforce. These streamlines help visualize how different AOTE settings impact the car’s performance and stability.

**Figure 11.**
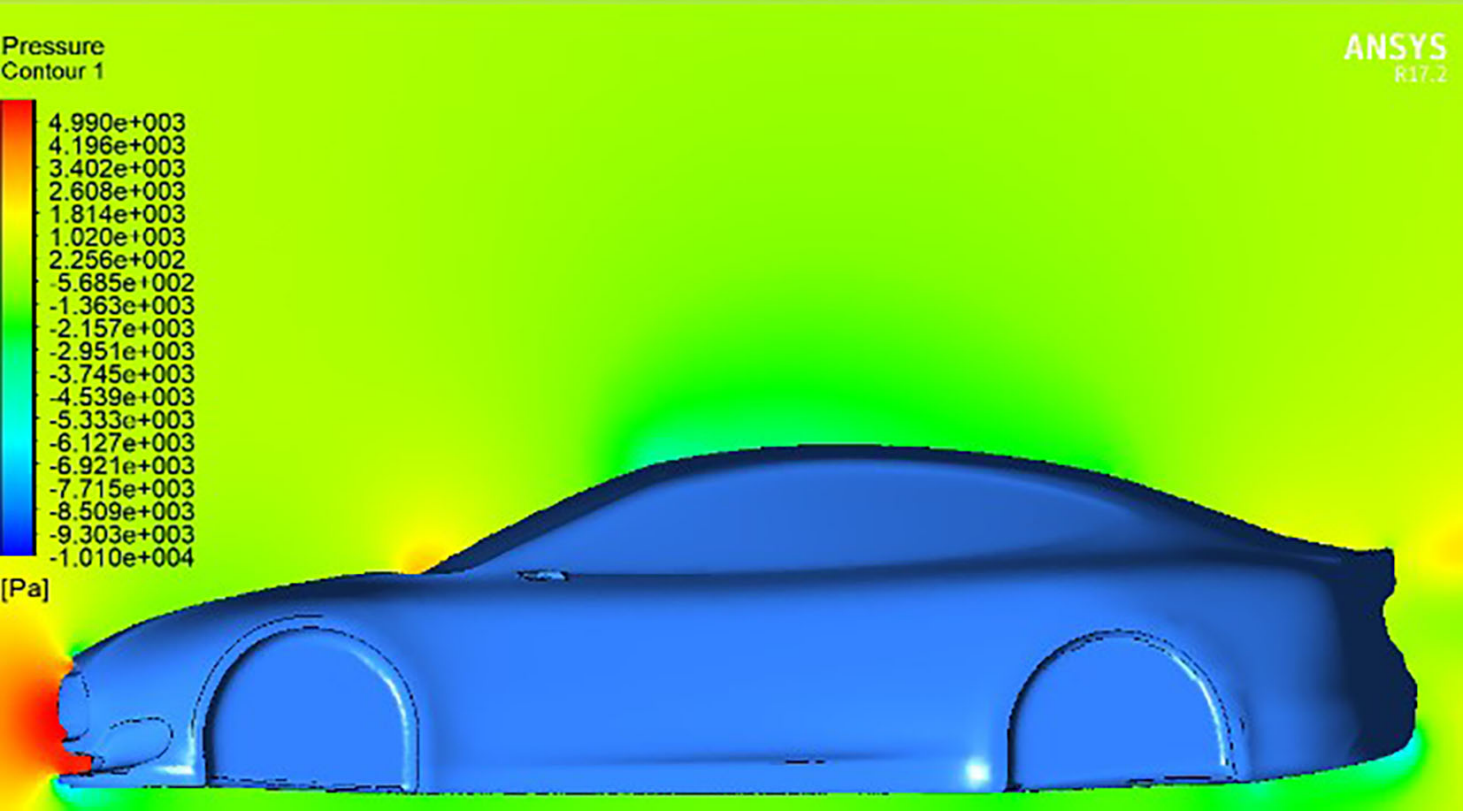
Pressure contour for car model.

**Figure 12.**
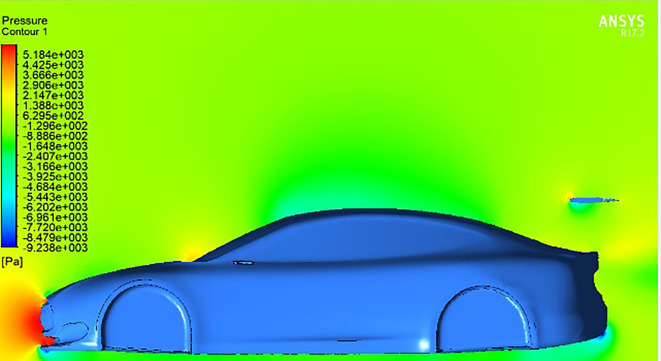
Pressure contour for car model and spoiler at 0 AOTE.

**Figure 13.**
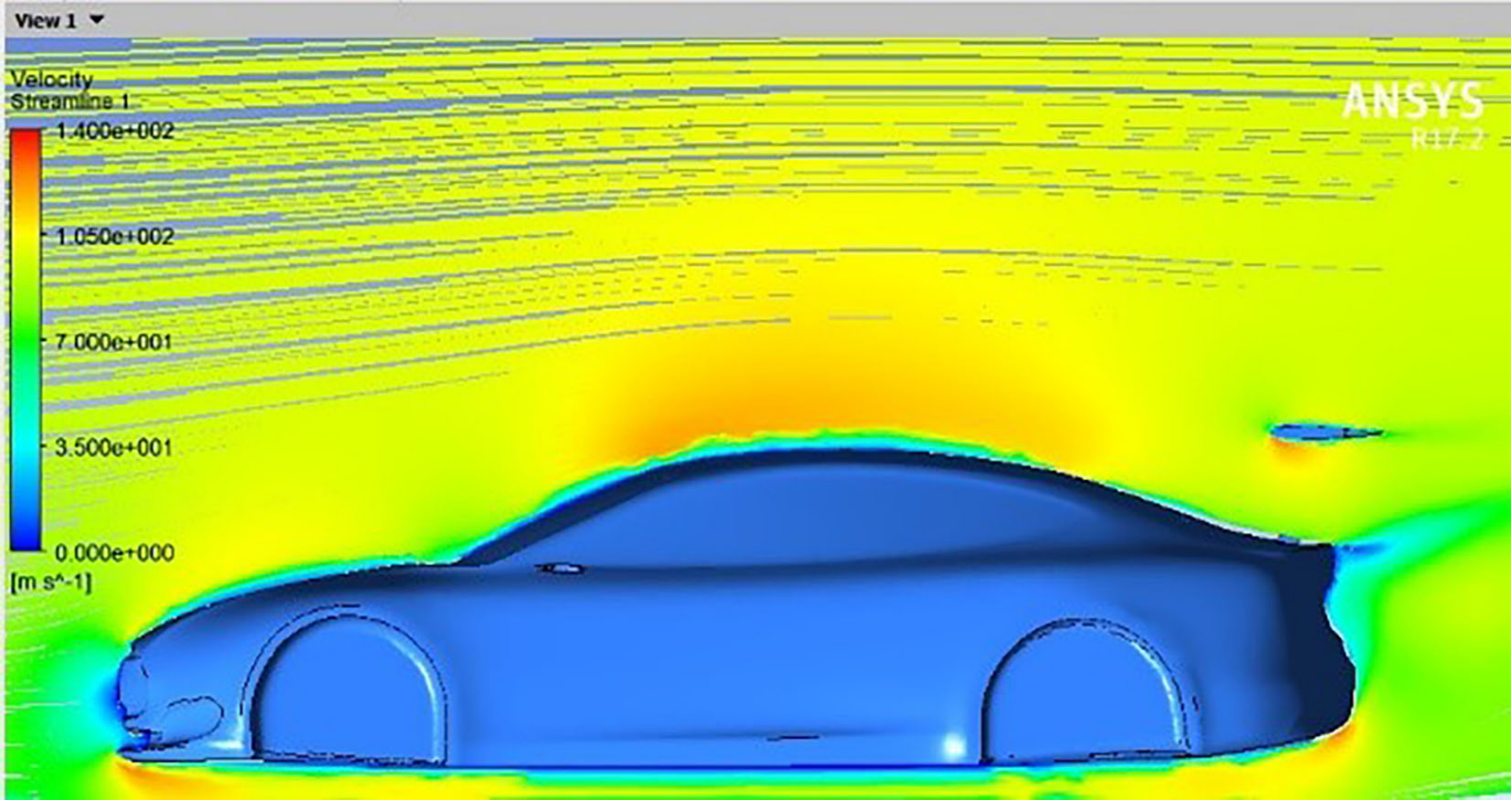
Streamline for car model and spoiler at 0 AOTE.

The illustrations provide a captivating exploration of the aerodynamic complexities surrounding a car’s wings. Initially, in the absence of a moving wing Figure 11, the car experiences a distribution of air pressure, mainly concentrated on the front bumper with slight effects on the windshield near the wipers. With the introduction of a rear wing with a trailing edge equal to zero degrees, as shown in Figure 12, we notice that a clear concentration of pressure appears in the front part of the spoiler, which affects the increase in downforce. This unexpected observation challenges the traditional assumptions of this type of airfoil, where no lift or pressure force is generated when the angle is zero. Figure 13 reveals the reason for this, as it reveals that the dynamic air flow interacts with the front part of the spoiler at an angle instead of colliding with the spoiler horizontally, which leads to an increase in downforce.

**Figure 14.**
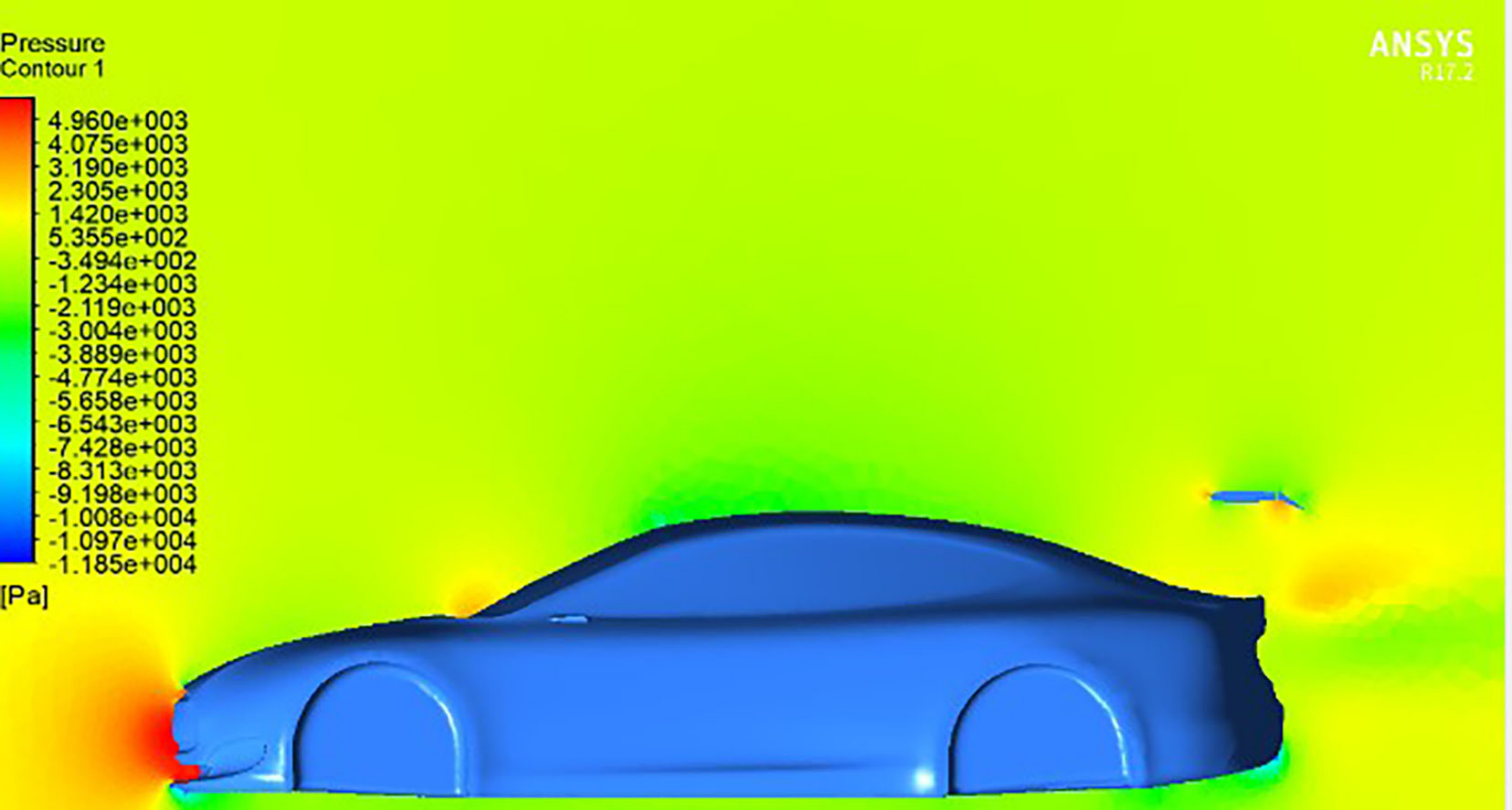
Pressure contour for car model and spoiler at negative AOTE.

**Figure 15.**
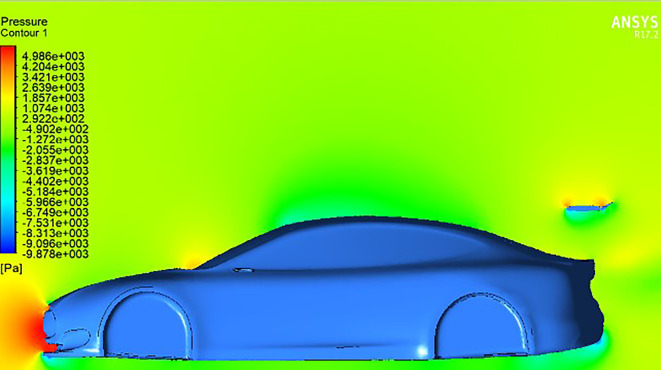
Pressure contour for car model and spoiler at positive AOTE.

And in Figure 15, a higher trailing edge angle results in a noticeable concentration of pressure on the trailing edge of the wing, resulting in a significant increase in downforce and a consequent enhancement of the vehicle’s aerodynamic stability. Figure 14. Here, the downward motion of the trailing edge angle redirects airflow down the trailing edge of the wing, generating lift forces. This aerodynamic phenomenon introduces a counterforce that reduces the overall downforce.

In summary, the figures underscore the essential function of spoilers in aerodynamic control. Through visual examination, it becomes evident how alterations in spoiler angles can profoundly influence airflow patterns, leading to enhanced performance and manoeuvrability. This emphasizes the significance of carefully adjusting spoiler configurations to optimize vehicle dynamics and efficiency.

**Lift force chart and table**

**Figure 16.**
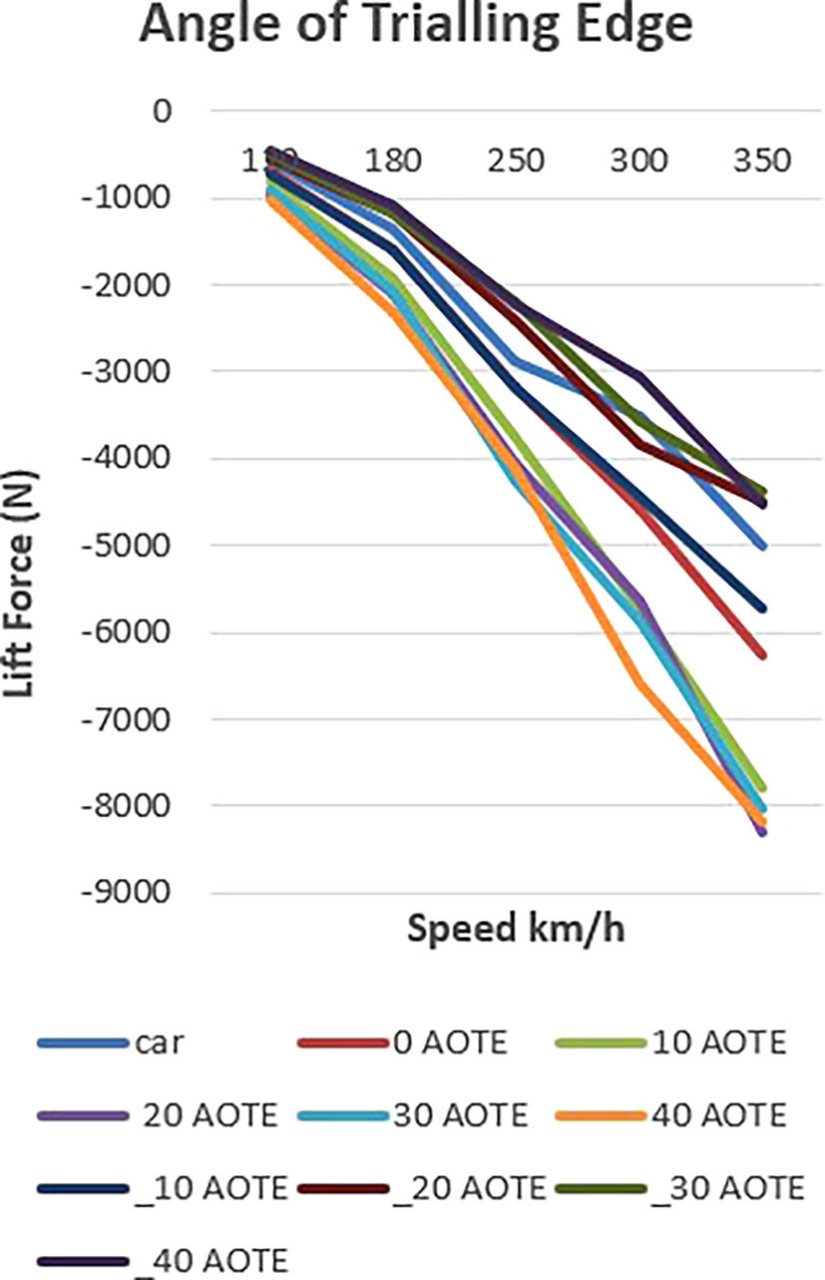
Down force with different AOTE.

The relationship between speed, angle of trailing edge (AOTE), and the resulting lift force is illustrated in Figure 16 and Table 4. The data points represent measurements taken at specific speeds with corresponding AOTE values, demonstrating how these factors influence the lift force generated. The results highlight the significant impact of AOTE adjustments on vehicle aerodynamics, particularly at higher speeds.

**Table 4.** Lift force with different of AOTE table.

		120 (KM/H)	180 (KM/H)	250 (KM/H)	300 (KM/H)	350 (KM/H)
**Car**	FL (N)	-573.2	-1360	-2886	-3505	-5015
	CL	-0.368	-0.388	-0.427	-0.36	-0.379
**0 AOTE**	FL (N)	-701.4	-1587.1	-3182	-4584	-6264
	CL	-0.437	-0.44	-0.456	-0.457	-0.458
**10 AOTE**	FL (N)	-809.12	-1932	-3746	-5723	-7793
	CL	-0.503	-0.534	-0.537	-0.57	-0.57
**20 AOTE**	FL (N)	-952	-2100	-4060	-5631	-8298
	CL	-0.58	-0.575	-0.578	-0.5553	-0.6
**30 AOTE**	FL (N)	-901	-2086	-4254	-5867	-8043
	CL	-0.56	-0.568	-0.6	-0.575	-0.58
**40 AOTE**	FL (N)	-1016	-2312	-4110	-6609	-8183
	CL	-0.561	-0.62	-0.45	-0.564	-0.58
**-10 AOTE**	FL (N)	-722.22	-1593	-3166	-4449	-5725
	CL	-0.45	-0.44	-0.402	-0.443	-0.419
**-20 AOTE**	FL (N)	-545.5	-1180	-2394	-3840	-4487
	CL	-0.337	-0.324	-0.341	-0.38	-0.326
**-30 AOTE**	FL (N)	-481	-1178	-2197	-3561	-4369
	CL	-0.295	-0.321	-0.31	-0.345	-0.315
**-40 AOTE**	FL (N)	-467	-1070	-2219	-3057	-4530
	CL	-0.294	-0.3185	-0.28	-0.3105	-0.3

**Figure 17.**
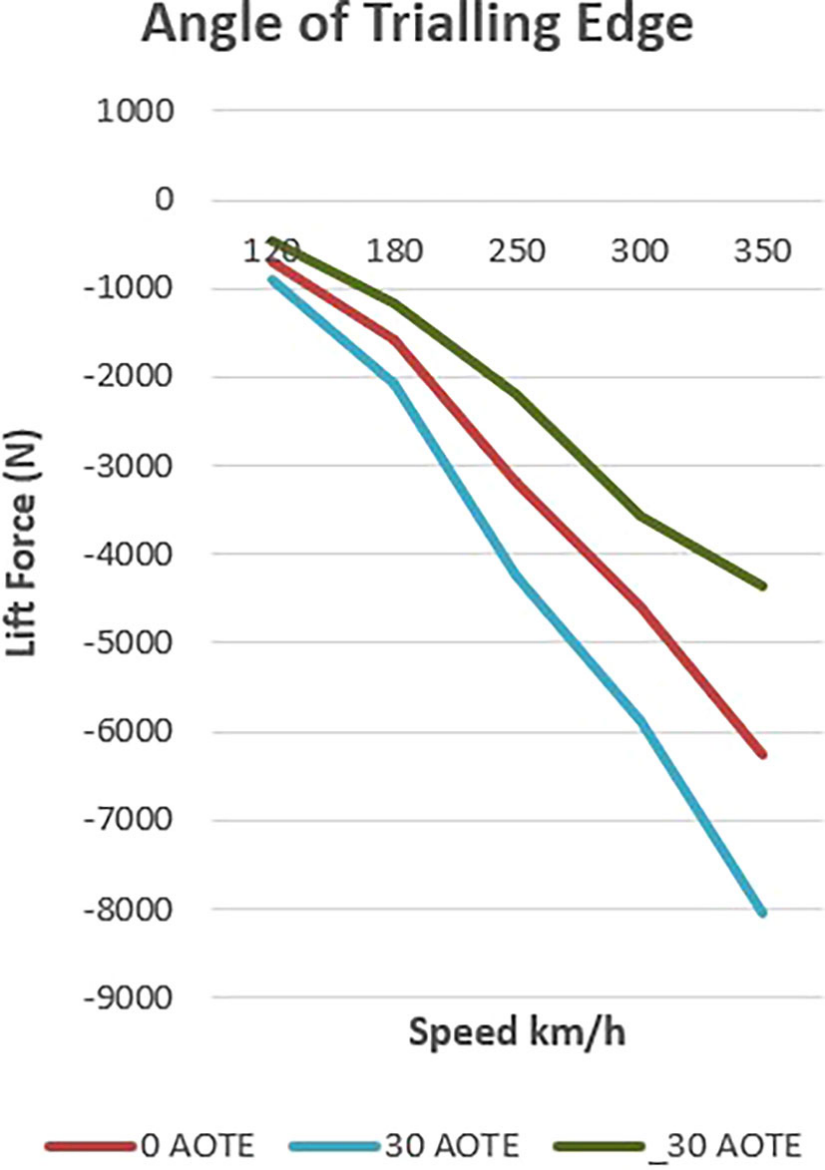
Down force at (-30, 0, 30) AOTE.

The presented Figure 17 delineates the influence of controlled downforce manipulation on vehicle dynamics, specifically focusing on lift and downforce. In the baseline scenario, the vehicle exhibits a progressive increase in downforce with rising velocity, reflecting its inherent design characteristics.

Upon the introduction of a spoiler mechanism and subsequent adjustments to its configuration, a discernible alteration in lift and downforce profiles is observed. This controlled manipulation of aerodynamic forces enables a targeted optimization strategy aimed at augmenting both stability and efficiency metrics.

Lowering the spoiler configuration results in a reduction of downforce, thereby mitigating lift and enhancing overall aerodynamic efficiency. This reduction translates into diminished aerodynamic drag, consequently improving the vehicle’s efficiency profile. Conversely, elevating the spoiler configuration yields an amplification of downforce, concurrently bolstering vehicle stability, particularly during high-speed manoeuvres where lateral stability is critical.

Notably, the depiction of lift and downforce values as negative underscores their respective orientations. This negative representation signifies their downward and upward forces, respectively. Thus, even amid downforce reduction, stability remains preserved, mitigating potential risks associated with compromised stability, such as lateral instability or loss of traction.

In summary, the chart elucidates the efficacy of controlled downforce manipulation in optimizing vehicle dynamics. Through strategic adjustments to spoiler configuration, this study presents a scientifically grounded approach to concurrently enhancing stability and efficiency without compromising safety or performance criteria in vehicular engineering.

**Figure 18.**
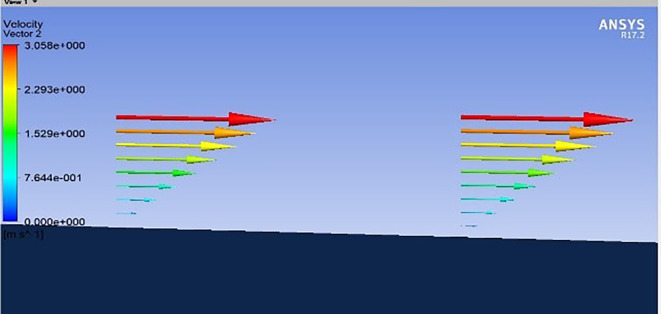
Boundary layer.

**Figure 19.**
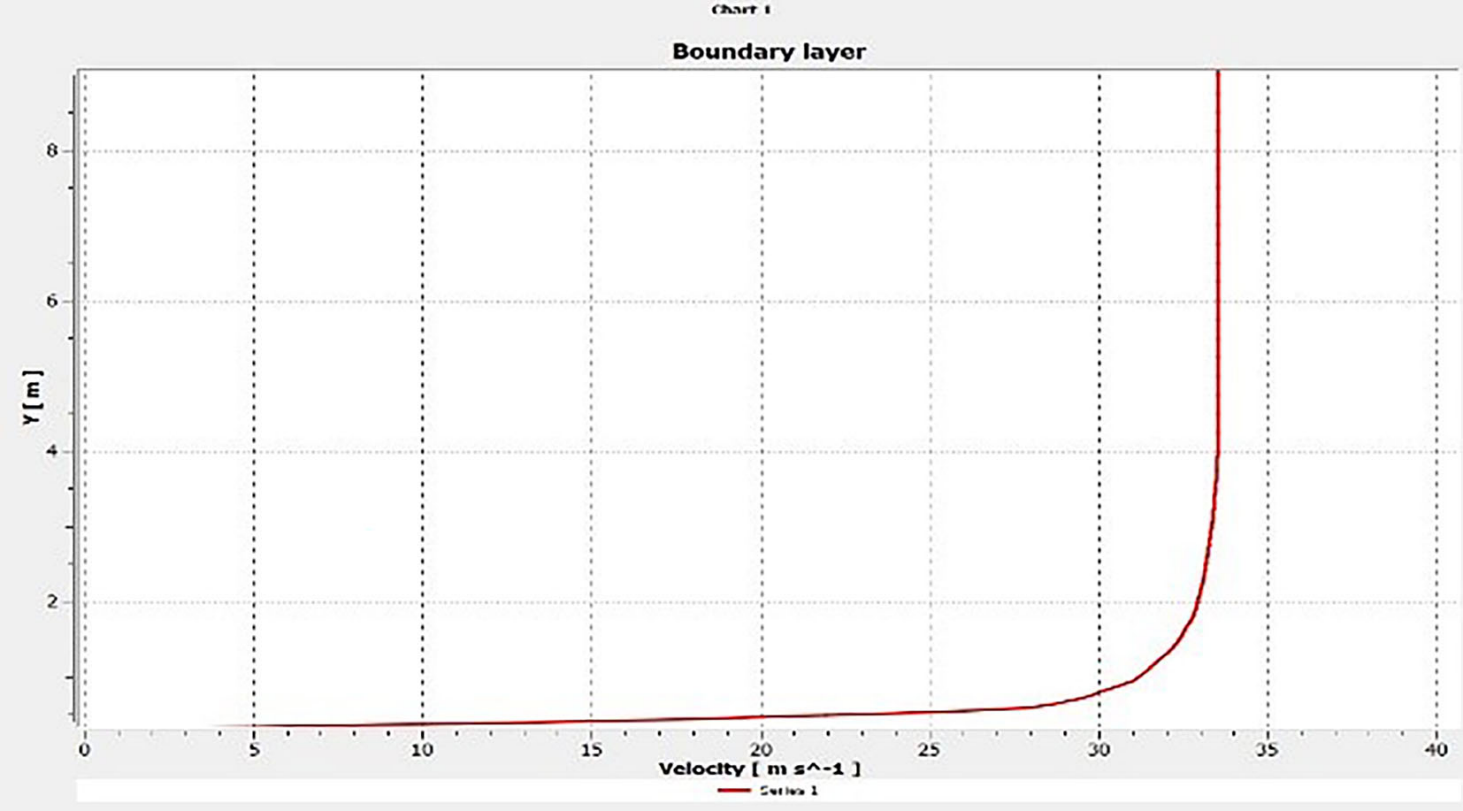
Boundary layer.

 Drag Force

**Figure 20.**
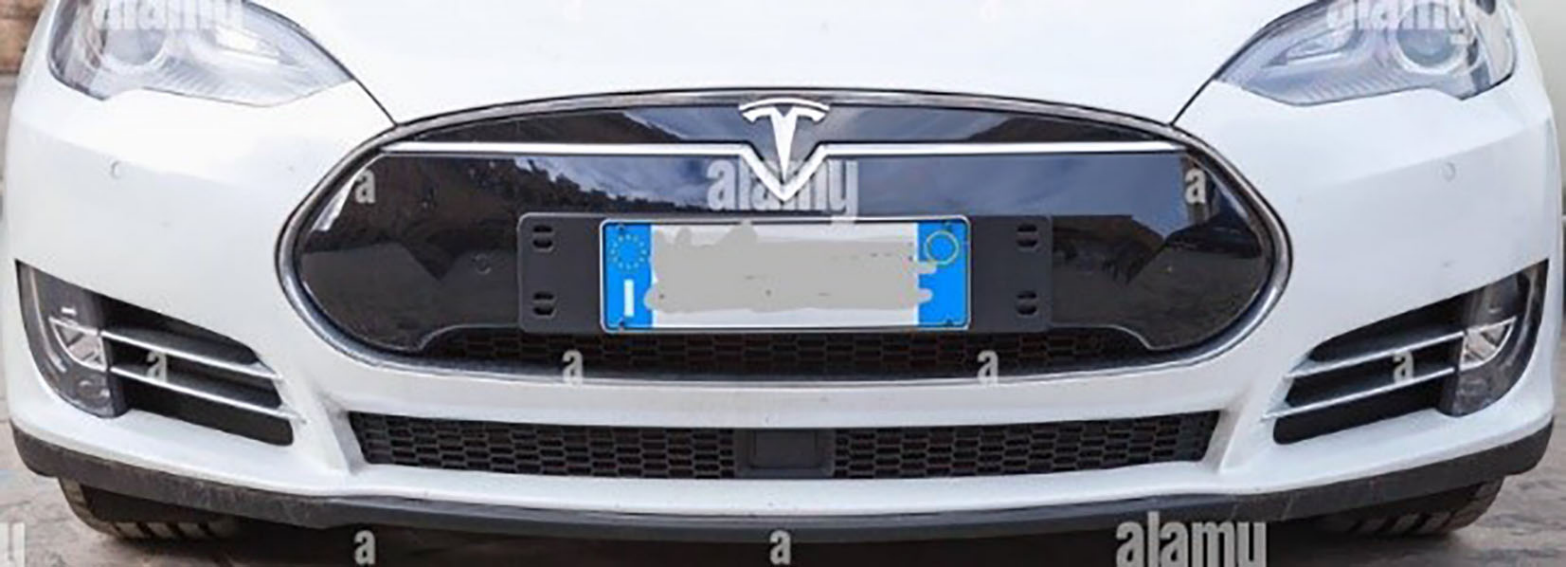
Tesla S model 2015 front bumper design.

**Figure 21.**
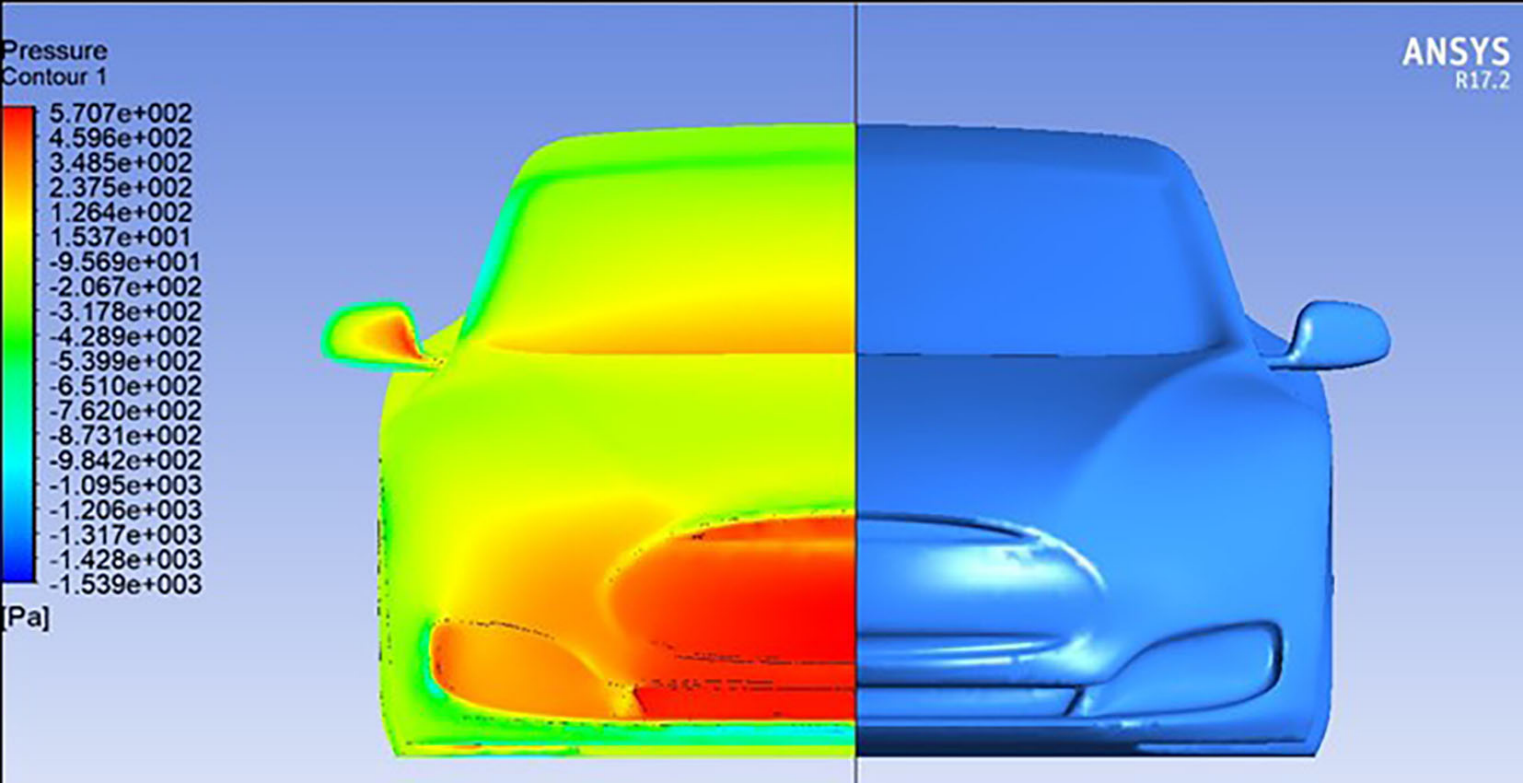
Pressure contour.

The simulation results yielded a drag force coefficient of **0.256** for the car, which represents a **6% deviation** from Tesla’s officially reported coefficient of **0.24.** ^9^ This discrepancy can be attributed to two primary factors:

1. **Mesh Refinement**: The computational mesh used in the simulation, while adequate for initial analysis, could be further refined to enhance accuracy. A higher-resolution mesh would likely produce aerodynamic data more closely aligned with real-world measurements.
2. **Front Bumper Design**: The actual Tesla Model S incorporates perforations in the front bumper, as depicted in Figure 20, Figure 21 which are designed to reduce drag. These features were not replicated in the simulation, resulting in a higher simulated drag coefficient.

To address this discrepancy, future simulations should focus on improving mesh resolution and incorporating detailed design elements, such as the front bumper perforations. These adjustments would enable the simulation results to more accurately reflect the aerodynamic performance reported by Tesla. The drag coefficients for various spoiler configurations are presented in Table 5.

**Table 5.** Drag coefficient with different of AOTE table.

	CD
Tesla car	0.24
Simulated car	0.256
Car with spoiler at -30 AOTE	0.278
Car with spoiler at 0 AOTE	0.2823
Car with spoiler at 30 AOTE	0.306

The simulations were conducted in compliance with Tesla’s testing guidelines, which specify a test speed of **70 mph** for determiningaerodynamic coefficients. This ensures that the simulation conditions are consistent with real-world testing protocols, providing a reliable basis for comparison.

**Spoiler with and without holder effect**

The placement of a rear spoiler on a vehicle, whether with or without stands (feet), ^10, 11^ can affect its aerodynamic performance. This study investigates this phenomenon by analysing lift force data obtained from spoilers with and without stands. Comparing the results highlights notable differences in aerodynamic behaviour based on stand placement. This underscores the importance of stand configuration in rear spoiler design and its implications for optimizing vehicle aerodynamics.

**Figure 22.**
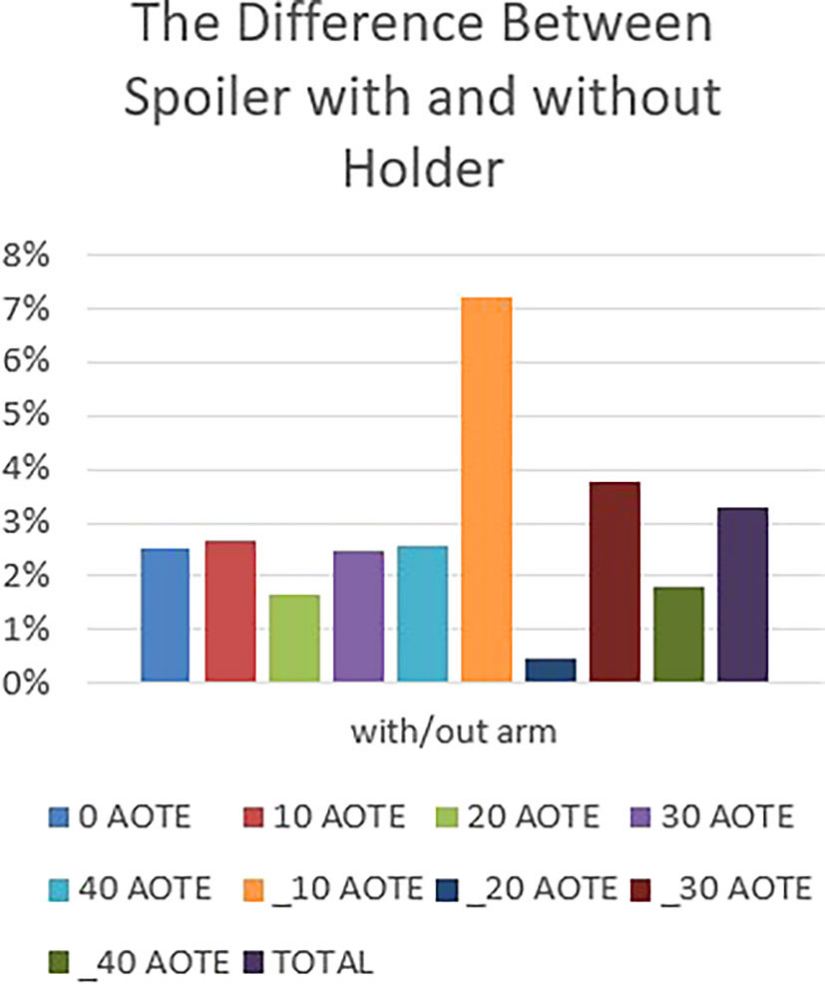
The difference between spoiler with and without holder.

The graph in Figure 22 indicates a clear difference between the results obtained for the spoiler with or without a holder (stand, feet, etc.). This variance underscores the significant influence of the holder’s design, shape, and positioning on aerodynamics. It emphasizes the importance of carefully considering these factors in holder design to optimize aerodynamic performance and ensure effective integration with the vehicle’s overall aerodynamic profile.

## Conclusion

The project focused on enhancing vehicle performance through the application of airfoils as spoilers with a movable trailing edge has provided valuable insights into automotive dynamics. The key findings are as follows:

(1) **Dynamic downforce control**: The use of airfoils with a movable trailing edge in spoiler design allows for real-time adjustment of downforce. This enables precise tuning of aerodynamic properties, enhancing vehicle stability and handling in various driving conditions.
(2) **Enhanced stability and handling**: By dynamically increasing or decreasing downforce, the vehicle maintains optimal grip and balance, especially during high-speed driving and cornering. This results in improved safety, better handling, and increased driver confidence, reducing the risk of skidding and loss of control.
(3) **Improved acceleration**: The ability to reduce downforce when it is not needed, such as during straight-line acceleration, minimizes the negative impact of excessive downforce on speed. This leads to improved acceleration performance, allowing the vehicle to achieve faster and more efficient speed increases without compromising stability.
(4) **Feasibility of integration**: The project has demonstrated that integrating this advanced aerodynamic feature into existing vehicle systems is feasible without extensive modifications. This practical aspect facilitates easier adoption and implementation within the automotive industry.
(5) **Future research and development**: Further research is recommended to optimize the control algorithms and fully explore the benefits of this technology under a wider range of conditions. Continued development will focus on maximizing performance gains, ensuring reliability, and assessing long-term durability.

In conclusion, the application of airfoils as spoilers with movable trailing edges represents a significant advancement in vehicle performance enhancement. This innovative approach allows for precise control of downforce, leading to improved stability, handling, and acceleration. The findings provide a solid foundation for future developments, highlighting the potential for widespread application of this technology to enhance vehicle performance, efficiency, and safety.

### Note

Some of the simulated data has been attached to a public database that you can view. ^12^

### Ethics and consent

Ethical approval and consent were not required for this study, as it did not involve human participants, animal subjects, or sensitive data. The research focused solely on computational simulations and analysis of aerodynamic performance using publicly available data and software tools.

## Data Availability

The data can be accessed at: https://doi.org/10.5281/zenodo.14602400. ^12^ Data are available under the terms of the Creative Commons Zero “No rights reserved” data waiver (CC0 1.0 Public domain dedication).

## References

[1] Smith A Johnson B : Aerodynamic design of automotive spoilers. *Journal of Vehicle Engineering.* 2018;25(3):123–140.

[2] Patel S Garcia M : Optimizing automotive spoiler design for improved aerodynamic performance. *International Journal of Vehicle Design.* 2021;35(2):145–162.

[3] Thompson C : Effects of spoiler configurations on speed and handling. *International Journal of Automotive Performance.* 2019;12(2):78–92.

[4] Chen X : Stability enhancement and rollover prevention with automotive spoilers. *Journal of Vehicle Safety.* 2017;22(4):321–340.

[5] Green R : Environmental considerations in automotive spoiler design. *Journal of Sustainable Transportation.* 2022;40(1):56–75.

[6] Lee H Kim J : Design integration of spoilers in vehicles for improved efficiency. *International Journal of Automotive Engineering.* 2019;29(4):321–336.

[7] Menter FR : Zonal two equation k-ω turbulence models for aerodynamic flows. *AIAA Paper.* 1993;93–2906.

[8] Menter FR : Two-equation eddy-viscosity turbulence models for engineering applications. *AIAA J.* 1994;32(8):1598–1605.

[9] Tesla: *Five slippery cars enter a wind tunnel.* Tesla;2024, August 16. Reference Source

[10] Youssef MI : Using CFD analysis to investigate the appropriate height of the rear spoiler on a car. *Zenodo.* 2022, August. 10.5281/zenodo.7036018

[11] Kozak J : Car design as a new conceptual solution and CFD analysis in purpose of improving aerodynamics. *Journal of Automotive Engineering.* 2014;15(3):45–60. Reference Source

[12] Karaki A : Enhancing vehicle performance through the application of airfoils as spoilers with movable trailing edge (Ansys.Products.17.2.Win64). *Zenodo.* 2025. 10.5281/zenodo.14602400 PMC12171717 40529636

