# Enhancing vehicle performance through the application of airfoils as spoilers with movable trailing edge

**DOI:** 10.12688/f1000research.160307.2

**Published:** 2025-06-09

**Authors:** Ahmad Karaki, Mohammad Abu Sirreya, Majdi Zalloum, Husein Amro

**Affiliations:** 1 mechanical engineering, Palestine Polytechnic University, Hebron, Palestinian Territory

**Keywords:** CFD, Simulation, Vehicle Dynamics, Spoiler, Trailing Edge.

## Abstract

**Background:**

Vehicle safety and stability are critical in the automotive industry, with aerodynamics playing a key role in enhancing these attributes. Spoilers, when effectively designed, can significantly influence airflow, downforce, and lift. This study investigates the aerodynamic performance of spoilers modeled as airfoils with adjustable trailing edges, aiming to dynamically control aerodynamic forces and improve vehicle stability and performance.

**Methods:**

Computational Fluid Dynamics (CFD) simulations were conducted using ANSYS Fluent® to analyze the impact of varying trailing edge angles (AOTE) on aerodynamic forces. A detailed Tesla vehicle model was created in CATIA™, and simulations were performed across a speed range of 120–350 km/h. The Shear Stress Transport (SST) *k–ω* turbulence model was employed to ensure accurate flow prediction. A wind tunnel domain and grid independence validation were used to ensure numerical reliability. Boundary conditions included velocity inlets, pressure outlets, and no-slip wall boundaries.

**Results:**

Adjusting the trailing edge angle produced significant variations in lift and downforce. At an angle of 30°, the negative lift (downforce) increased by up to 36%. At 0°, it increased by up to 17%. During acceleration phases, the controlled generation of positive lift improved aerodynamic efficiency, yielding a total lift increase of up to 15%. The simulated drag coefficient was 0.256, differing by 6% from Tesla’s reported value of 0.24, primarily due to mesh refinement level and geometric simplifications.

**Conclusions:**

This study demonstrates that a spoiler with a movable trailing edge can significantly enhance vehicle handling, acceleration, and aerodynamic stability by actively modulating lift and downforce. The findings support the integration of active aerodynamic control systems in vehicle design. Future research will focus on control system development and experimental validation under real-world driving conditions.

## Introduction

Automotive spoilers, once primarily associated with high-performance sports cars and racing vehicles, have become a common sight on a wide range of automobiles. These devices, typically mounted on the rear of a car, have gained popularity among car enthusiasts for their purported ability to enhance vehicle performance, stability, and fuel efficiency. This study delves into the effect of spoilers on car performance to provide a comprehensive understanding of their impact.

Spoilers, as aerodynamic appendages, are designed to modify the airflow around a vehicle. They can take various forms, including lip spoilers, wing spoilers, and even roof spoilers, each designed with distinct aerodynamic objectives. Some enthusiasts believe that the addition of a spoiler to a car can improve its speed, cornering capabilities, and fuel economy. However, the efficacy of these modifications depends on factors such as the design, size, and installation of the spoiler, as well as the specific characteristics of the vehicle it is attached to.

This study aims to systematically investigate the performance effects of spoilers with **movable trailing edges**, providing a detailed analysis of their influence on lift, downforce, and drag. Unlike previous work that often focuses on static or fixed-geometry spoilers, this research explores the **dynamic modulation of spoiler geometry** as a method of achieving real-time aerodynamic control. CFD simulations are used to quantify performance gains and validate the design approach.

Given the increasing emphasis on vehicle efficiency and stability in modern automotive design, a deeper understanding of spoiler aerodynamics is essential. This work contributes to that understanding by demonstrating how adaptive aerodynamic elements can be employed to enhance performance across a wide range of operating conditions.

This study embarks on a comprehensive exploration of the effects of spoilers on vehicle performance, using a combination of CATIA for design and ANSYS for computational fluid dynamics (CFD) simulations to provide a holistic understanding.

## Literature review

Spoilers, once the hallmark of high-performance racing vehicles, are now commonly integrated into a wide range of passenger cars. This widespread adoption has prompted increased interest in understanding their function beyond aesthetics, particularly in improving vehicle aerodynamics, stability, and energy efficiency.

Smith and Johnson ^1^ examined spoiler configurations and their influence on airflow separation and pressure distribution, emphasizing their role in increasing downforce and minimizing turbulence-induced drag. Patel and Garcia ^2^ utilized Computational Fluid Dynamics (CFD) simulations to analyze spoiler-induced pressure gradients and flow detachment, offering quantitative insights into optimizing aerodynamic performance through design refinement.

Complementing these computational studies, Thompson et al. ^3^ performed experimental assessments on various spoiler geometries, demonstrating how design changes directly influence vehicle dynamics, including acceleration, braking efficiency, and lateral stability. Chen et al. ^4^ expanded on this by analyzing the role of spoilers in vehicle rollover prevention. Their findings confirmed that properly configured aerodynamic devices contribute to improved safety under high-speed maneuvering by adjusting vertical load distribution.

In the context of sustainable design, Green et al. ^5^ investigated the ecological impact of spoiler integration, focusing on the trade-offs between aerodynamic gains and energy consumption. Concurrently, driver-centric studies ^6^ explored how perceived aesthetics and functional performance jointly influence spoiler adoption, especially in consumer vehicles.

Modern aerodynamic research has increasingly incorporated software tools like MATLAB and ANSYS to simulate and validate control mechanisms for aerodynamic elements. These platforms enable iterative design of adaptive systems aimed at achieving optimal flow control, energy savings, and dynamic response under varied driving conditions.

Despite these advances, most existing work centers around **static or passive spoiler systems**—devices with fixed geometry that are optimized for a narrow range of operating conditions. There remains a noticeable gap in the literature concerning **actively controlled, dynamically adjustable spoiler systems**, particularly those with **movable trailing edges** capable of responding in real time to changing aerodynamic demands.

This study addresses that gap by proposing and analyzing an **active spoiler configuration featuring a movable trailing edge**, designed using high-fidelity CAD tools and simulated under realistic driving speeds (120–350 km/h). Unlike previous works, which optimize spoiler effects at fixed positions, the present approach demonstrates how **real-time modulation of the trailing edge angle** can enhance lift and downforce control. This active aerodynamic concept aligns with emerging trends in adaptive vehicle design, contributing both to performance improvement and driving safety under dynamic conditions.

## Airfoil

Airfoil is a shape designed to generate lift when air flows over it. The shape is typically found in cross-sections of wings, blades, and other surfaces that move through air, such as aircraft wings, helicopter rotor blades, propellers, and wind turbines.

**Figure 1.**
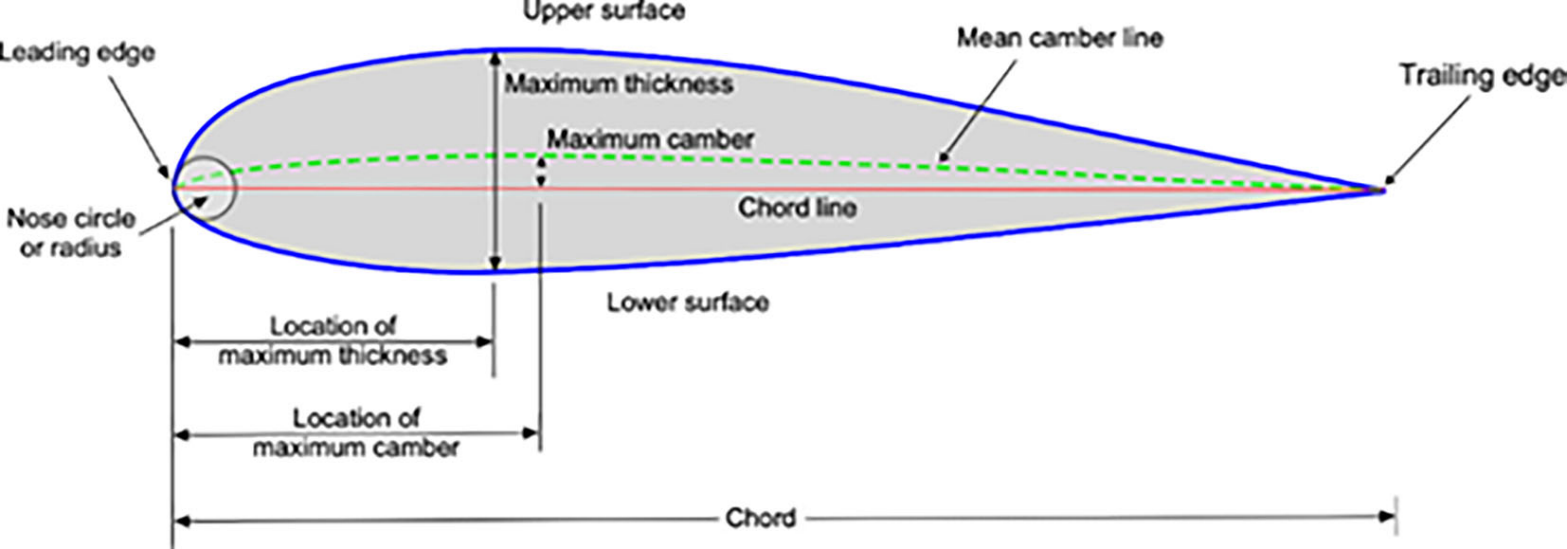
Airfoil geometry.

Figure 1 presents graphical depictions of key aerodynamic features associated with an airfoil, offering insights into its geometry. The following elements are typically illustrated:

(1) **Camber line**:
  The camber line represents the curve defining the maximum distance between the upper and lower surfaces of the airfoil. It characterizes the camber or curvature of the airfoil. This line is crucial in determining the lift characteristics of the airfoil, as it influences the distribution of pressure around the surface.
(2) **Chord line**:
  The chord line is a straight line connecting the leading edge to the trailing edge of the airfoil. It serves as a fundamental reference for aerodynamic analysis.
  The angle of attack, a parameter crucial to lift generation, is typically measured with respect to the chord line.
(3) **Thickness**:
  The thickness of the airfoil is the maximum distance between the upper and lower surfaces, perpendicular to the chord line. This dimension contributes to the overall aerodynamic characteristics and structural considerations of the airfoil.
(4) **Trailing edge**:
  The rear edge of an airfoil is where the airflow separated by the Leading Edge regions and where the essential control surfaces are located.

### Airfoil group

Understanding the aerodynamic characteristics of various airfoil shapes is essential for the design and performance optimization of aircraft wings and vehicle spoilers. Airfoils, the cross-sectional profiles of wings or spoilers, are designed to generate lift efficiently while minimizing drag. Table 1 provides a detailed comparison of different airfoil groups, highlighting their geometric parameters and aerodynamic coefficients.

**Table 1.** Airfoil group classification with geometric and aerodynamic data.

Group A					
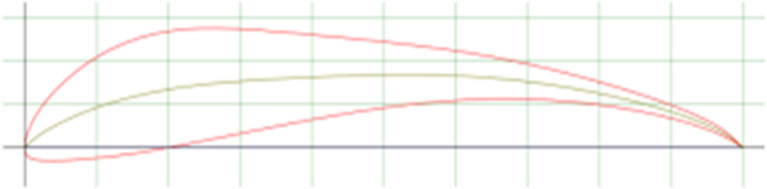					
Name	Thick	Camber	Chord	CD	CL
**S1223RTL**	13.5	8.3	0.3 m	0.047	1.019
**GOE243**	14.2	9.2	0.3 m	0.052	1.135
**FX73-CL3-152**	15.2	8	0.3 m	0.057	0.985
**USA32AIRFOIL**	14.7	9.3	0.3 m	0.068	1.146

Group B					
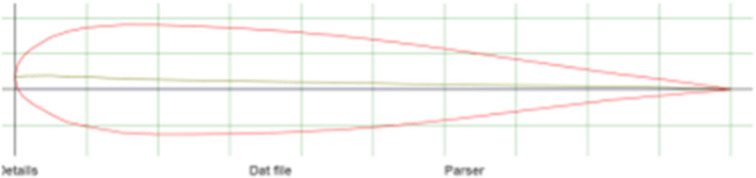					
Name	Thick	Camber	Chord	CD	CL
**NREL5801**	13.5	3.7	0.3 m	0.027	0.457
**EPPIER434**	13.3	4.3	0.3 m	0.028	0.531
**AH63-IC-127\24**	12.7	3.8	0.3 m	0.025	0.47
**AH93-K-130\15**	13.1	3.5	0.3 m	0.025	0.433

Group C					
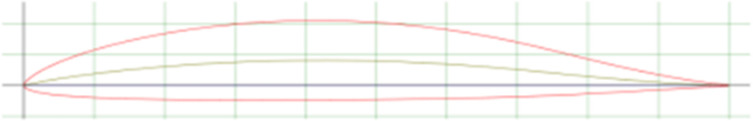					
Name	Thick	Camber	Chord	CD	CL
**DoEing737 root**	15.4	0.2	0.3 m	0.027	0.024
**OAF139**	13.7	0	0.3 m	0.024	0
**-E474**	14.1	0	0.3 m	0.025	0
**-EPPIER479**	16.6	0	0.3 m	0.028	0

Group D					
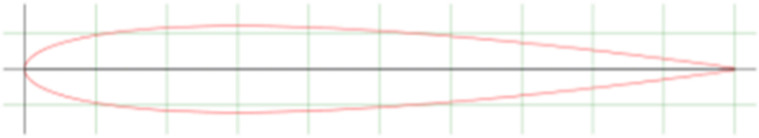					
Name	Thick	Camber	Chord	CD	CL
**NACA0012**	12	0	0.3 m	0.019	0
**-EPPLER EA(6)**	12	0	0.3 m	0.019	0
**-RAF30**	12.6	0	0.3 m	0.021	0
**-GOE 409**	12.7	0	0.3 m	0.021	0

This data was taken: at a speed of 120 km/h, altitude = 0 and wing area = 0.3 m ^2^.

Based on these comparisons, the **NACA0012** airfoil was chosen due to its well-documented symmetrical geometry and widespread acceptance in spoiler design applications. Its symmetrical profile ensures the lift force approaches zero at neutral angles of attack and exhibits minimal sensitivity to changes in airflow velocity, which is advantageous for spoilers that require precise lift control during both upward and downward deflections.

The geometric symmetry of the NACA0012 enables accurate and predictable modulation of lift force as the spoiler’s trailing edge angle changes. This stability and repeatability in aerodynamic response are critical for optimizing spoiler performance, especially during dynamic vehicle maneuvers demanding precise lift adjustments.

### CATIA model

The spoiler shapes were modeled using CATIA software to visualize the effects of varying trailing edge angles on the spoiler profile:

**Figure 2.**
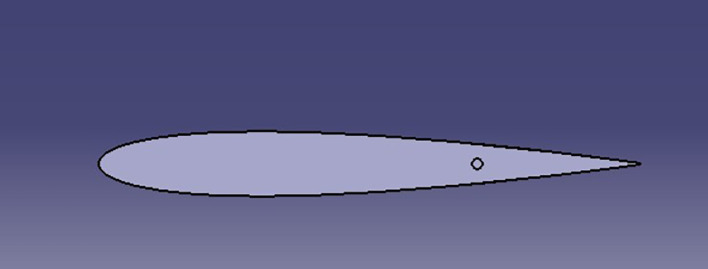
Spoiler model with trailing edge at 0° (neutral position).

**Figure 3.**
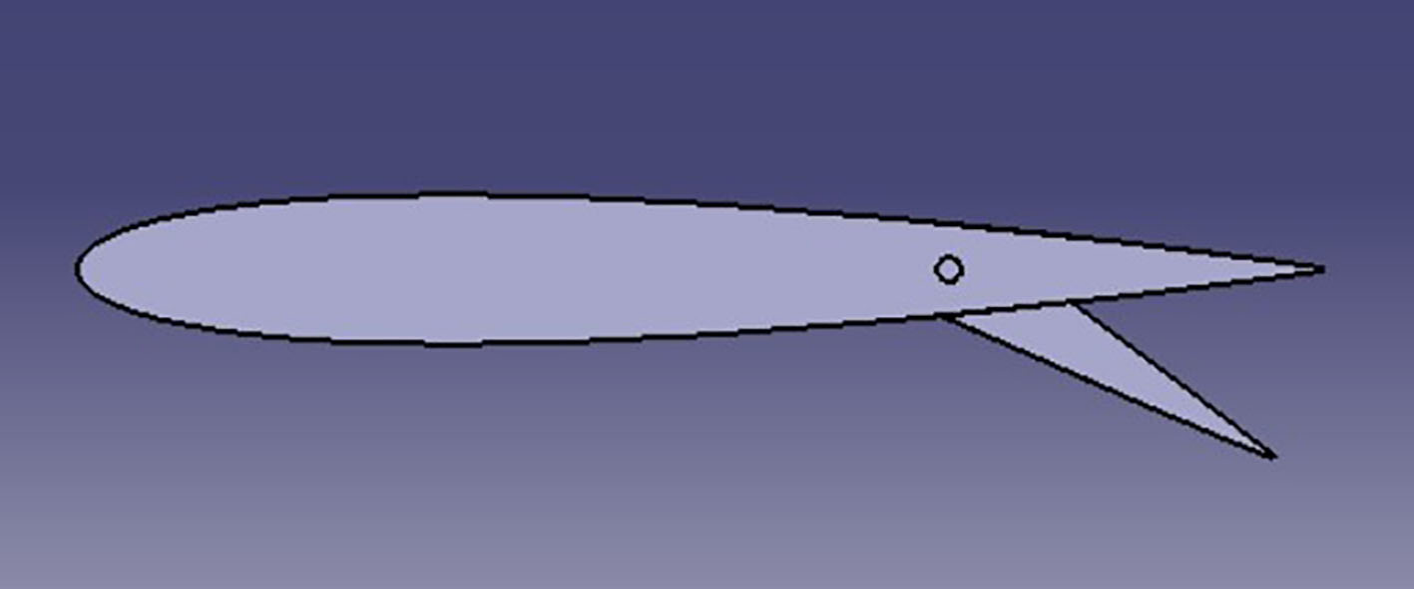
Spoiler model with trailing edge deflected negatively (downward angle).

**Figure 4.**
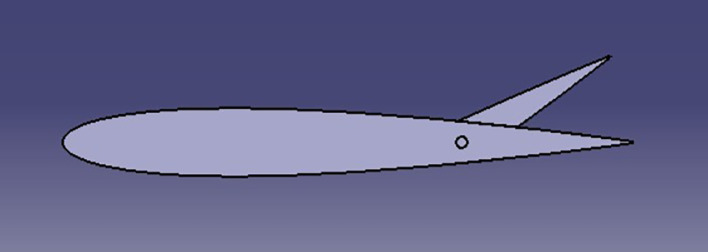
Spoiler model with trailing edge deflected positively (upward angle).

## Computational Fluid Dynamics (CFD)

It is a branch of fluid mechanics that uses numerical methods and algorithms to solve and analyze problems involving fluid flow. Fluid flow can occur in various contexts, such as air flow around an aircraft, water flow in rivers, heat transfer in pipes, and many others.

CFD simulations involve dividing the fluid domain into a grid or mesh of small elements and solving mathematical equations that govern fluid flow, heat transfer, and other related phenomena. These equations, which describe the conservation of mass, momentum, and energy, are solved iteratively to obtain a numerical solution that represents the behavior of the fluid.

In the automotive context, CFD is utilized to simulate and analyze the airflow around a car’s body and various components. Engineers can assess the impact of different design elements, such as spoilers, body shapes, and side mirrors, on aerodynamic performance. By virtually testing these components through CFD simulations, engineers can optimize designs to achieve goals like minimizing drag, maximizing downforce, and enhancing overall aerodynamic efficiency.

In summary, the integration of CFD in the automotive industry, particularly in the context of cars, allows engineers to optimize aerodynamic performance by virtually testing and refining designs. This approach significantly reduces the need for expensive and time-consuming physical experiments, leading to more efficient and effective vehicle designs.

### Turbulence model ( *SST k*- *ω Model*)

The Shear Stress Transport (SST) *k*- *ω* turbulence model, introduced by Menter, ^7^ is a widely used two-equation eddy-viscosity model known for its versatility in simulating complex flows. This model effectively combines the strengths of the *k*- *ω* and *k*- *ε* formulations. In the near-wall region, the *k*- *ω* formulation is employed, allowing the model to accurately capture boundary layer effects without requiring additional damping functions. In the free-stream region, the model transitions to *k*- *ε* behavior, addressing the sensitivity issues often associated with traditional *k*- *ω* models when dealing with free-stream turbulence properties.

One of the key advantages of the SST *k-ω* model is its robust performance in flows with adverse pressure gradients and separation, making it particularly suitable for simulating the complex airflow patterns around vehicle spoilers. However, the model tends to overpredict turbulence levels in regions with high normal strain, such as stagnation points or areas of strong acceleration. Despite this limitation, the SST *k-ω* model remains a preferred choice for aerodynamic simulations due to its overall accuracy and reliability. ^8^

**Figure 5.**
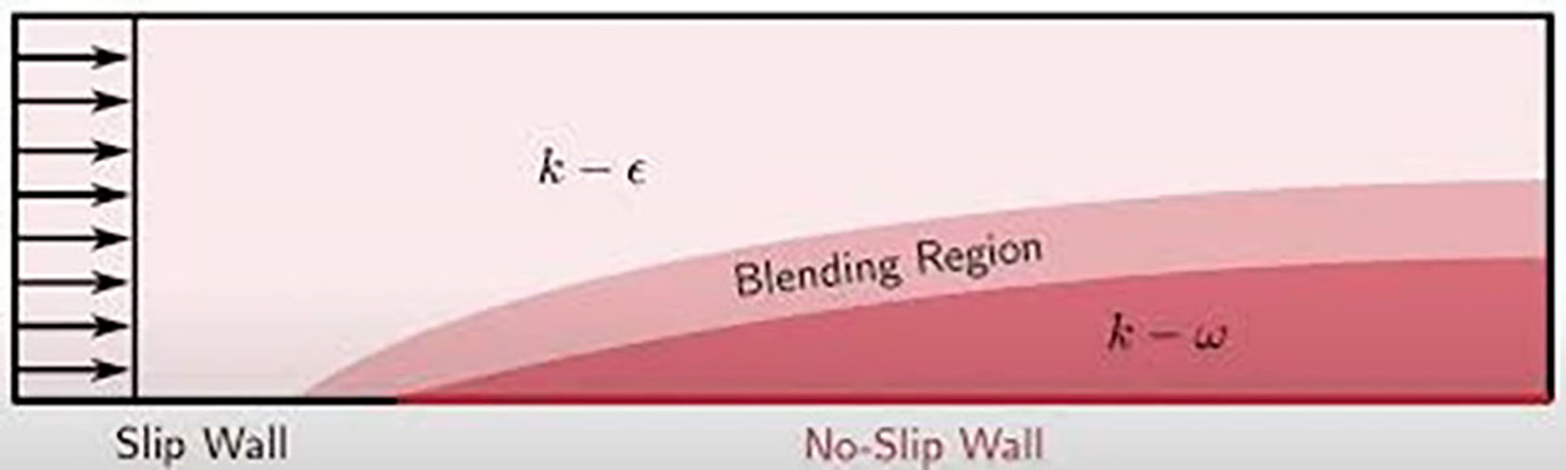
Turbulence model.

Kinematic Eddy Viscosity:

$$v_{T}=\frac{a_{1}k}{\max\left(a_{1}\omega ,SF_{2}\right)}$$ (1)

Where:

*a*_1_: Model constant, typically around 0.31.

***S***: Strain rate magnitude.

Turbulence Kinetic Energy:

$$\frac{\mathrm{\partial k}}{\mathrm{\partial t}}+U_{j}\frac{\mathrm{\partial k}}{\partial x_{j}}=P_{k}-\beta^{*}\mathrm{k\omega}+\frac{\partial }{\partial x_{j}}\left[\left(v+\sigma_{k}v_{T}\right)\frac{\mathrm{\partial k}}{\partial x_{j}}\right]$$ (2)

Specific Dissipation Rate:

$$\frac{\partial \omega }{\mathrm{\partial t}}+U_{j}\frac{\partial \omega }{\partial x_{j}}=\alpha S^{2}-\beta \omega^{2}+\frac{\partial }{\partial x_{j}}\left[\left(v+\sigma_{\omega }v_{T}\right)\frac{\partial \omega }{\partial x_{j}}\right]+2\left(1-F_{1}\right)\sigma_{\omega 2}\frac{1}{\omega }\frac{\mathrm{\partial k}}{\partial x_{j}}\frac{\partial \omega }{\partial x_{j}}$$ (3)

Where:

*k*: Represents the energy contained in turbulent eddies.

*P*: Production of *k*, usually due to velocity gradients.

*β* ^∗^: Model constant, controls the dissipation rate of *k* to *ω.*

*ω*: Specific dissipation rate.

*v*: Kinematic viscosity.

*σ*: Model constant, controls the turbulent diffusion of *k.*

*v* *_t_*: Turbulent viscosity.

Closure Coefficients and Auxiliary Relations:

$$F_{2}=\tanh\left[{\left[\max\left(\frac{2\sqrt{k}}{\beta^{*}\mathrm{\omega y}},\frac{500v}{y^{2}\omega }\right)\right]}^{2}\right]$$ (4)

$$P_{K}=\min\left(\tau_{\mathrm{ij}}\frac{\partial U_{i}}{\partial x_{i}},10\beta^{*}\mathrm{k\omega}\right)$$ (5)

$$F_{1}=\tanh\left\{{\left\{\min\left[\max\left(\frac{2\sqrt{k}}{\beta^{*}\mathrm{\omega y}},\frac{500v}{y^{2}\omega }\right),\frac{4\sigma_{\omega 2}k}{{\mathrm{CD}}_{\mathrm{k\omega}}y^{2}}\right]\right\}}^{4}\right\}$$ (6)

$${\mathrm{CD}}_{\mathrm{k\omega}}=\max\left(2\rho \sigma_{\omega 2}\frac{1}{\omega }\frac{\mathrm{\partial k}}{\partial x_{i}}\frac{\partial \omega }{\partial x_{i}},10^{-10}\right)$$ (7)

$$\alpha_{1}=\frac{5}{9},\alpha_{2}=0.44$$ (8)

$$\beta_{1}=\frac{3}{40},\beta_{2}=0.44$$ (9)

$$\beta^{*}=\frac{9}{100}$$ (10)

$$\sigma_{k1}=0.85,\sigma_{k2}=1$$ (11)

$$\sigma_{\omega 1}=0.5,\sigma_{\omega 2}=0.856$$ (12)

• **Near-Wall Treatment**: In the near-wall region, *F*1≈1 ensures that the *k*- *ω* model is used, which is more accurate for capturing the effects close to the wall.
• **Away from the Wall**: Further away from the wall, *F*1 decreases towards 0, blending towards the *k*- *ϵ* model, which is better suited for free-stream turbulence.
• **Turbulent Viscosity Adjustment**: *F*2 further refines the turbulent viscosity calculation to ensure smooth and accurate transitions between the near-wall and far-field regions.

## Ansys simulation and result analysis

This chapter presents the results of aerodynamic simulations conducted using ANSYS Fluent ^®^ software (ANSYS, Inc., Canonsburg, PA, USA). Two primary configurations were analyzed: a baseline car model without any modifications and a modified car model equipped with a spoiler featuring adjustable trailing edge angles (AOTE). The simulations were performed to evaluate the impact of spoiler adjustments on vehicle aerodynamics and stability.

The car model was designed using CATIA, and the computational domain was created to represent a wind tunnel. The simulations were conducted at various speeds, ranging from 120 km/h to 350 km/h, to assess the aerodynamic forces under different driving conditions. The Shear Stress Transport (SST) *k*- *ω* turbulence model was employed to capture the complex airflow patterns around the vehicle and spoiler.

### Car model

**Figure 6.**
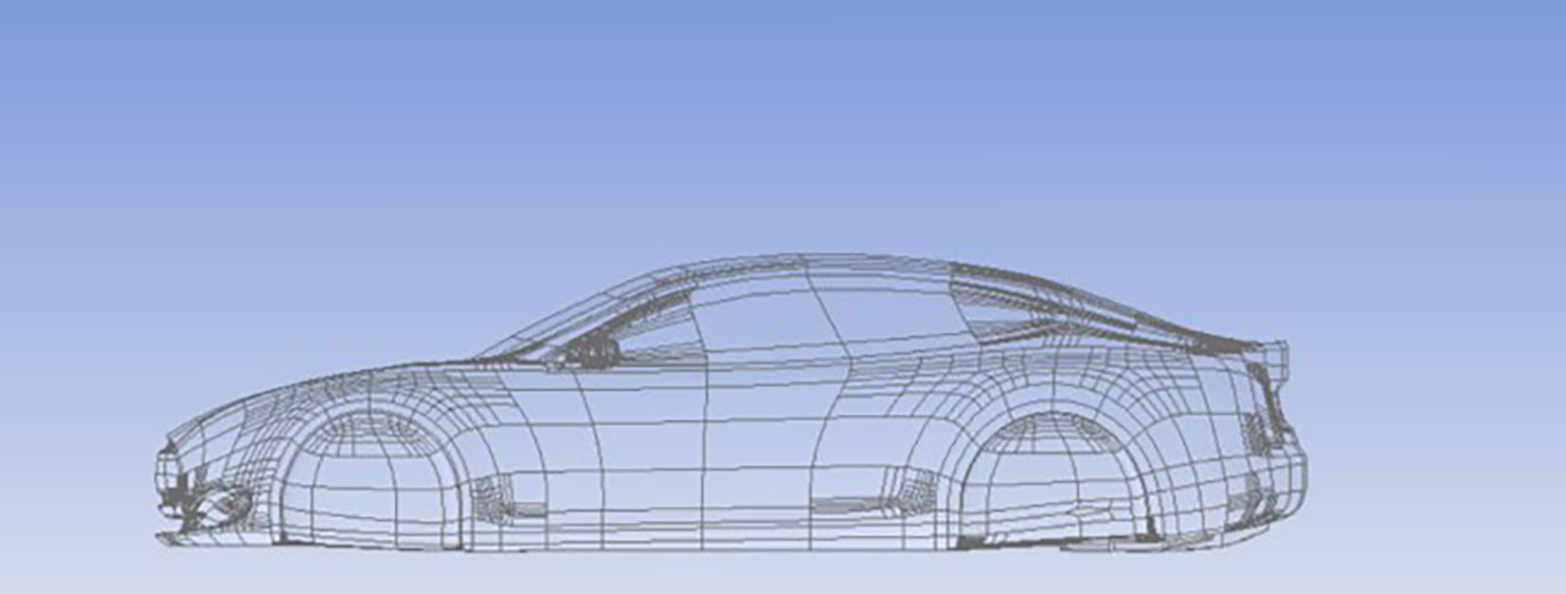
Car model designed in CATIA.

A simplified Tesla-inspired car model was designed using CATIA software, as shown in Figure 6.

### Car model with spoiler

The spoiler design utilizes the **NACA0012** airfoil profile, with dimensions of 120 cm length by 45 cm width. The spoiler features multiple trailing edge angles (AOTE) to investigate aerodynamic variations.

**Figure 7.**
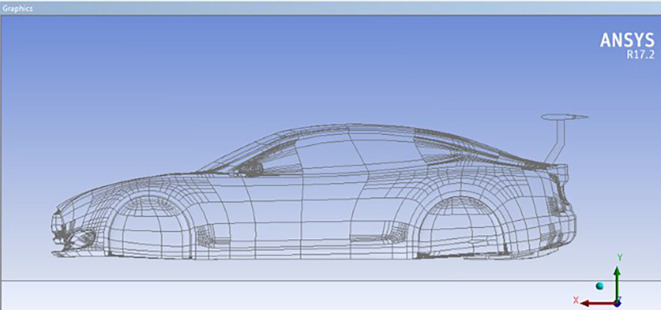
Car model equipped with adjustable trailing edge.

### Simulation Workflow

**Figure 8.**
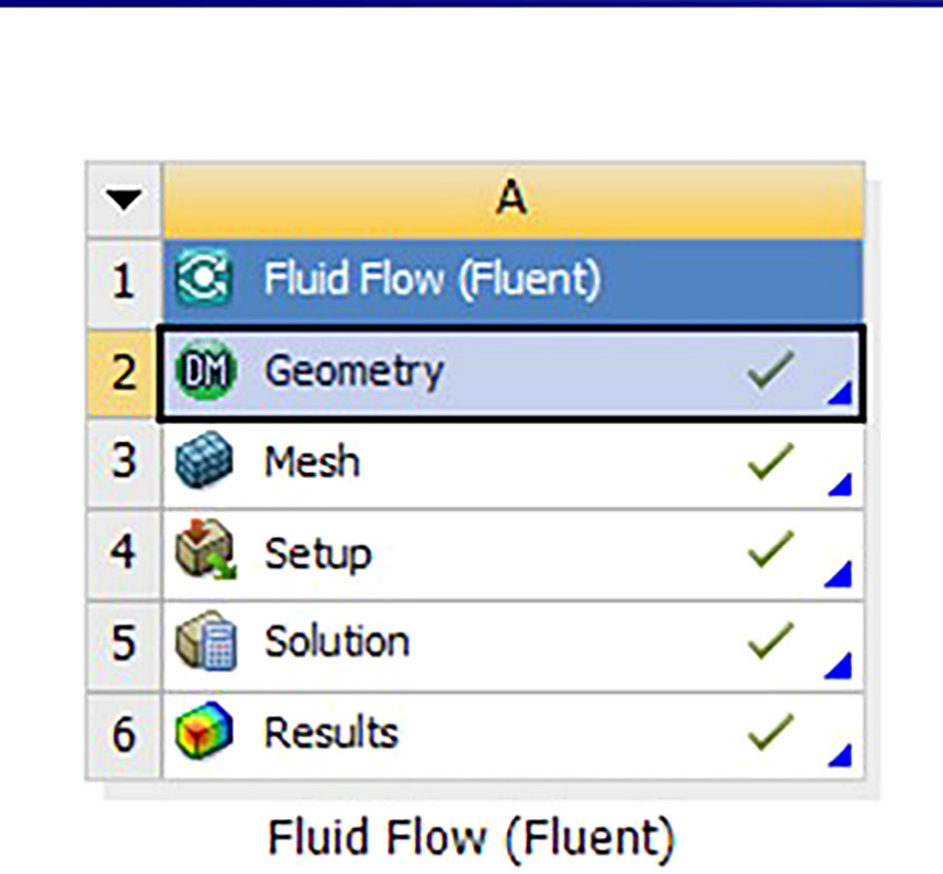
Simulation process workflow in ANSYS Workbench.

The ANSYS simulation process follows a structured sequence to ensure accurate results. Key steps include pre-processing, mesh generation, boundary condition setup, and turbulence model selection. The overall workflow within ANSYS Workbench is summarized in Figure 8.

**Figure 9.**
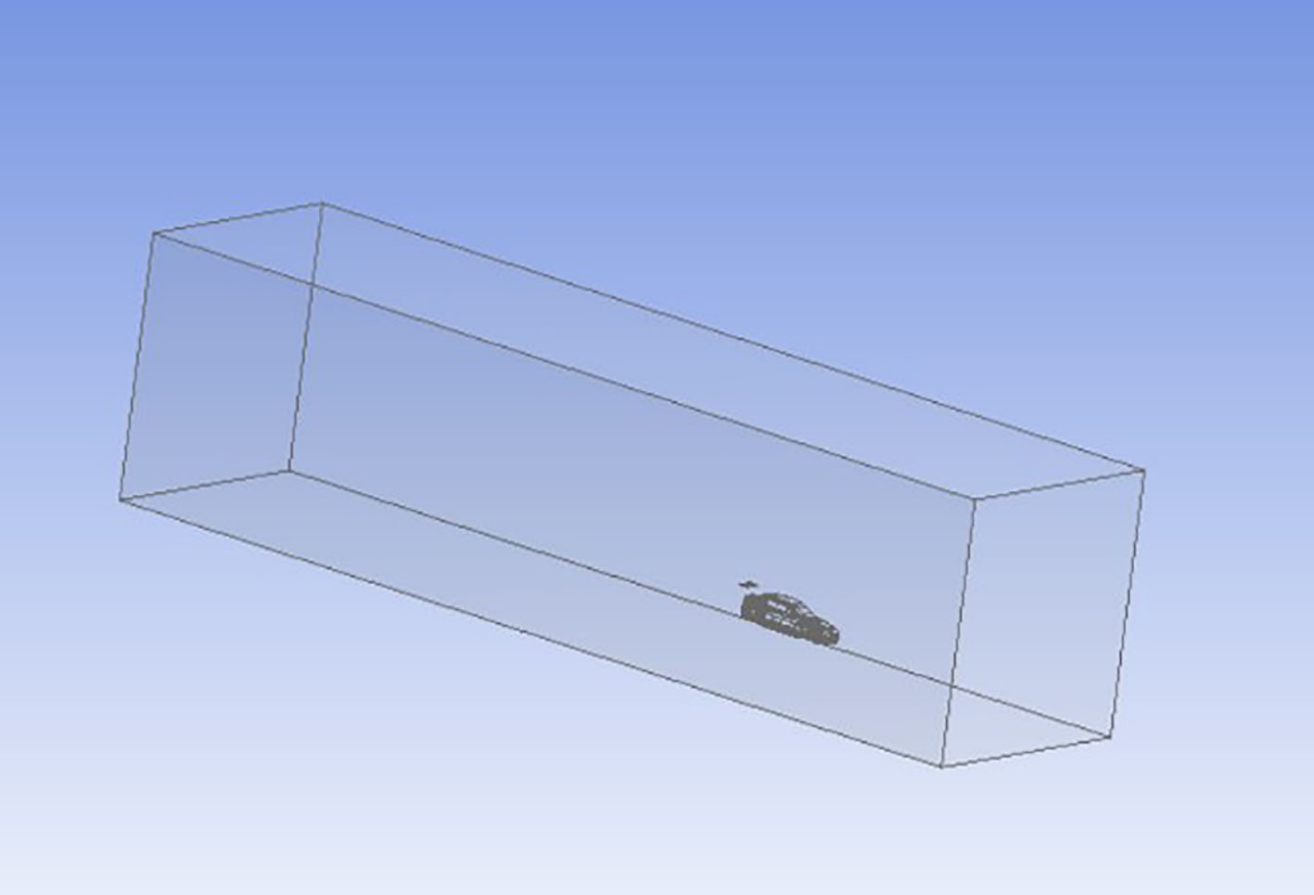
Computational domain enclosing the car model.

**Pre-processing **:

During pre-processing, the CATIA 3D car model is imported into ANSYS Geometry. A computational domain (“enclosure”) simulating the wind tunnel is created around the car. The enclosure dimensions are summarized in Table 2, and its 3D visualization is shown in Figure 9.

**Table 2.** Computational domain dimensions.

X	Y	Z
15 m	10 m	10 m
-25 m	-0.2 m	-10 m

**Meshing**:

The computational domain was discretized using a **polyhedral mesh** generated with ANSYS meshing tools. Local mesh refinement was applied around the entire vehicle surface, including the spoiler, to accurately capture flow gradients and aerodynamic phenomena across all critical regions.

The mesh employed a curvature-based size function with high smoothing and a medium relevance center to balance accuracy and computational efficiency. Key mesh parameters include:

• **Nodes:** 559,233
• **Elements:** 3,121,642
• **Minimum edge length:** 1.58 × 10 ^−5^ m

**Figure 10.**
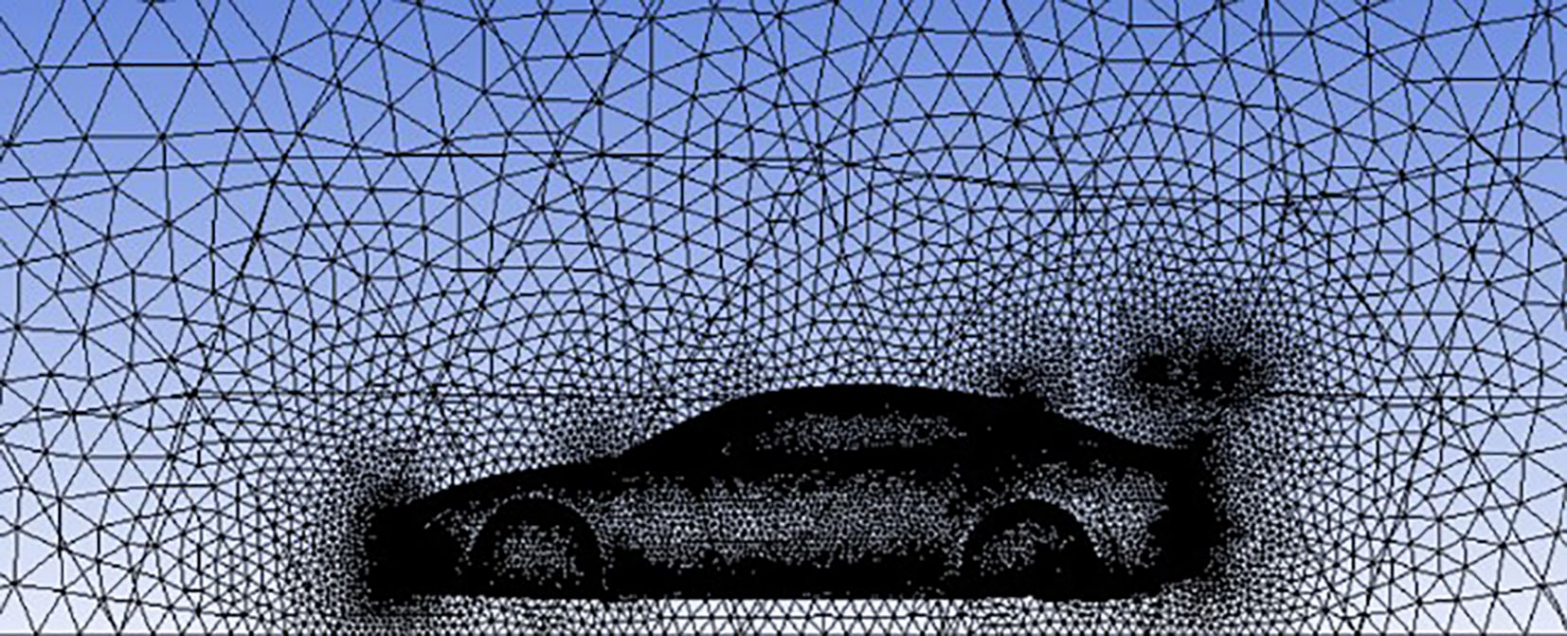
Meshed computational domain around the car model.

This mesh resolution ensures detailed representation of complex flow behavior around the vehicle. The meshed model is illustrated in Figure 10.

**Boundary conditions**:

Realistic airflow conditions were defined via boundary conditions as listed in Table 3. The inlet boundary is a velocity inlet with specified speeds of 120, 180, 250, 300, and 350 km/h, and a turbulence intensity of 5%.

**Table 3.** Boundary condition details.

Name	Boundary type	Boundary details
Inlet	Velocity-inlet	Normal speed, fore cases: 120 km/h, 180 km/h, 250 km/h, 300 km/h and 350 km/h. turbulence: medium (intensity = 5%).
Outlet	Pressure-outlet	Absolute
Wall	Wall	No slip
Symmetry	Symmetry	Half of enlister
Car	Wall	No slip

The outlet is set as a pressure outlet at atmospheric conditions. The car and spoiler surfaces apply no-slip wall conditions, while symmetry boundary conditions reduce computational requirements by simulating half the domain.

**Turbulence model**:

The final step in the setup process is selecting the turbulence model. The Shear Stress Transport (SST) *k*- *ω* model is chosen for its ability to accurately capture the complex airflow patterns around the vehicle, particularly in regions with adverse pressure gradients and flow separation.

### Result analysis and discussion

**Streamline and Pressure Contour Analysis:**

Following preprocessing and mesh generation, CFD simulations were executed using ANSYS Fluent to analyze the aerodynamic effects of varying **Angles of the Trailing Edge (AOTE)** on the vehicle’s performance. This section presents the results in the form of streamline plots and pressure contours, which offer critical insights into airflow behavior and the resulting aerodynamic forces.

**Figure 11.**
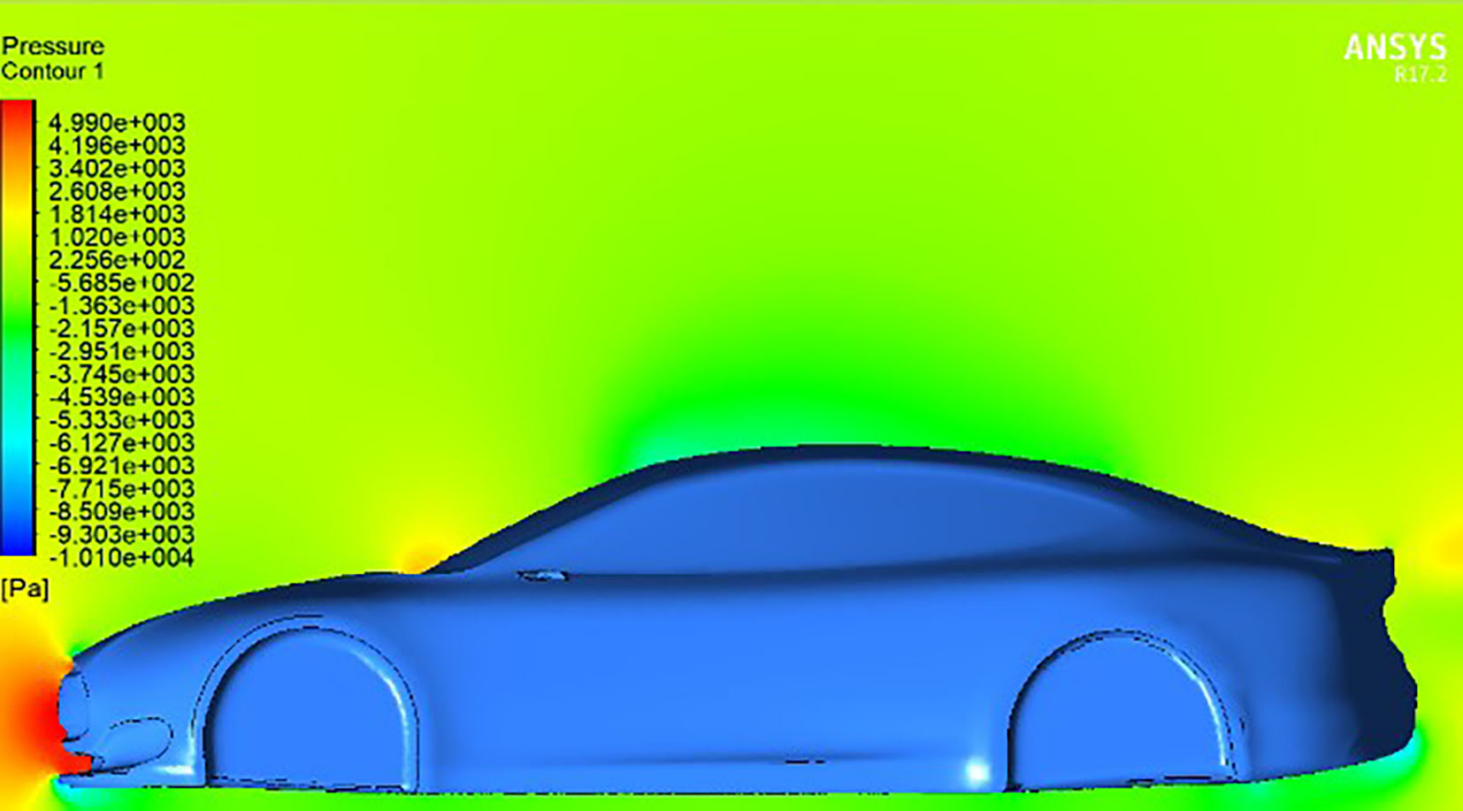
Pressure contour of the base car model without a spoiler.

Figure 11 shows the baseline pressure distribution around the car model without a spoiler. The majority of pressure is concentrated on the front bumper, with minor accumulation near the windshield region. This distribution reflects typical drag behavior experienced by streamlined bluff bodies at high speeds.

**Figure 12.**
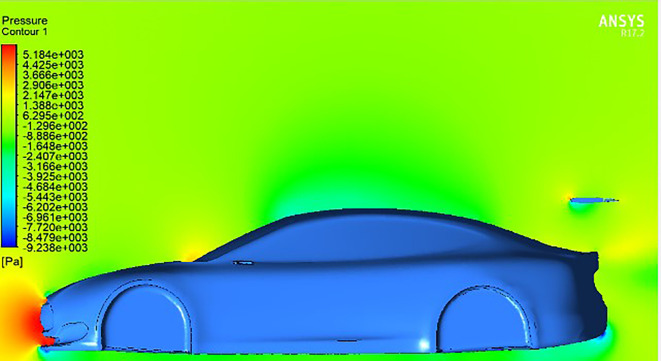
Pressure contour for the car with a spoiler at 0° AOTE.

The addition of a spoiler with **0° AOTE** ( Figure 12) leads to a significant pressure buildup on the spoiler’s leading edge, contributing to increased downforce. Although a symmetrical airfoil like **NACA0012** is expected to generate zero lift at 0° angle, the streamline analysis in ( Figure 15) reveals that airflow interacts with the spoiler at a slight angle due to vehicle-induced flow curvature. This results in an effective increase in downforce, contrary to theoretical expectations for neutral configurations.

**Figure 13.**
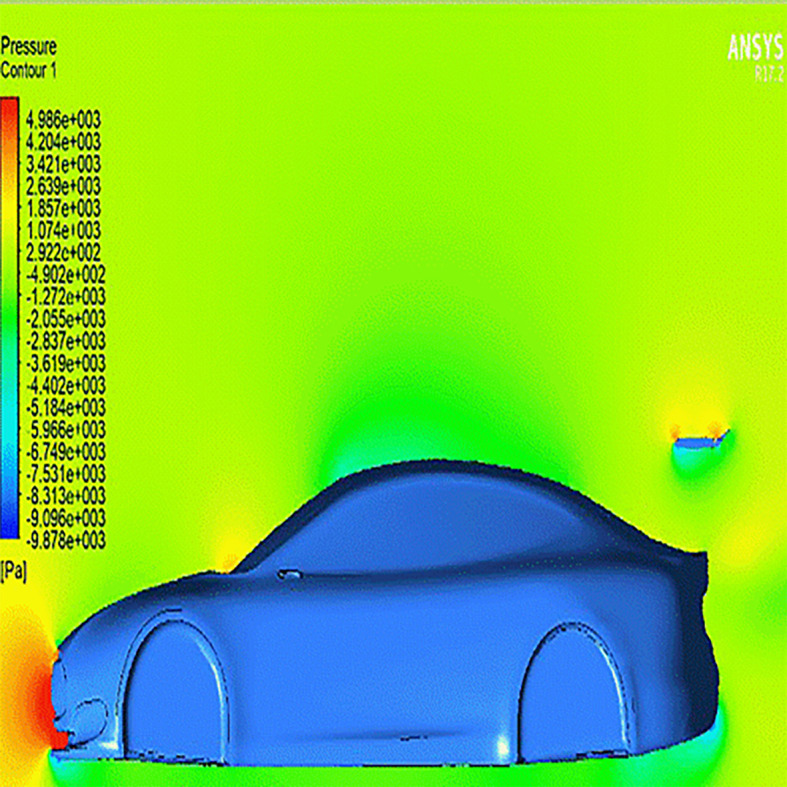
Pressure contour for spoiler at positive AOTE.

**Figure 14.**
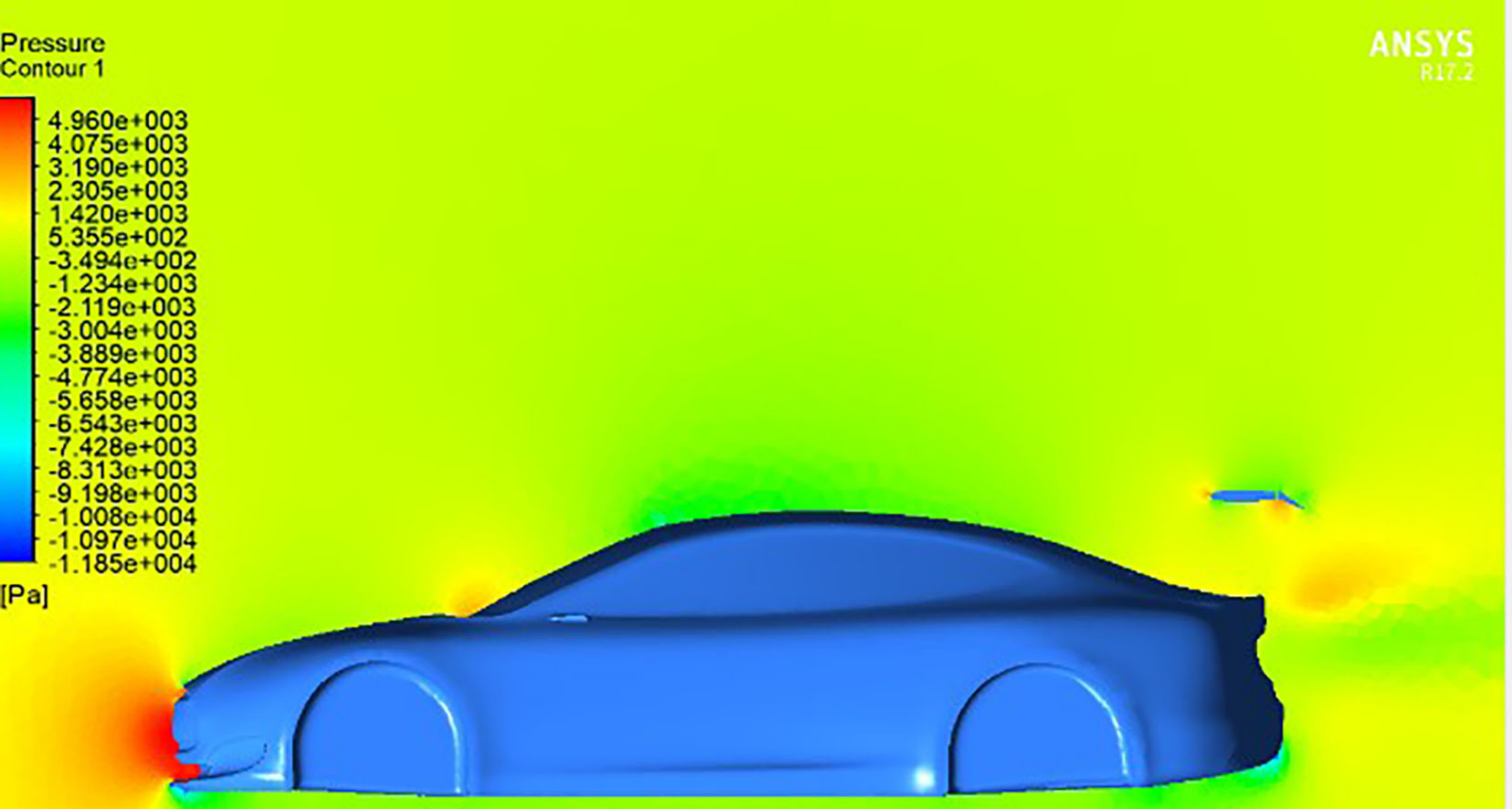
Pressure contour for spoiler at negative AOTE.

At **negative AOTE** settings ( Figure 14), the trailing edge deflects downward, causing the airflow to be redirected upward. This produces a counter-lifting effect that slightly reduces overall downforce. In contrast, at **positive AOTE** ( Figure 13), airflow is deflected more aggressively downward at the trailing edge, leading to high pressure concentration and a substantial increase in downforce. This configuration enhances rear-end grip and aerodynamic stability, particularly at higher velocities.

These variations in flow behavior demonstrate the dynamic aerodynamic response enabled by adjusting the spoiler’s trailing edge. By modifying the AOTE, the vehicle can fine-tune lift and downforce characteristics in real time, which is particularly beneficial for improving handling and performance under changing driving conditions.

**Figure 15.**
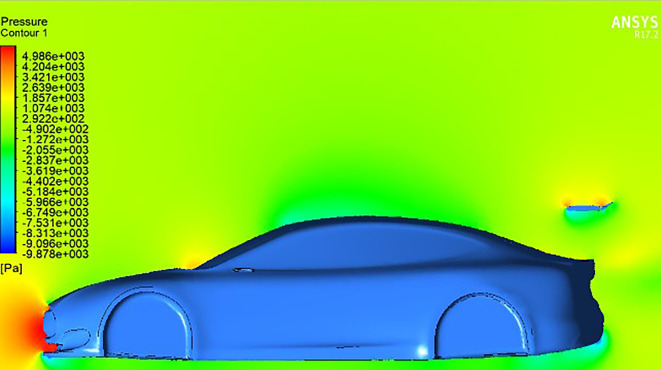
Streamlines for car with spoiler at 0° AOTE.

In summary, the streamline and pressure contour analysis emphasize the aerodynamic effectiveness of a movable trailing edge spoiler. Adjustments in the spoiler’s geometry can significantly influence airflow patterns and pressure distribution, resulting in measurable improvements in downforce and vehicle stability.

**Lift force variation with AOTE**

**Figure 16.**
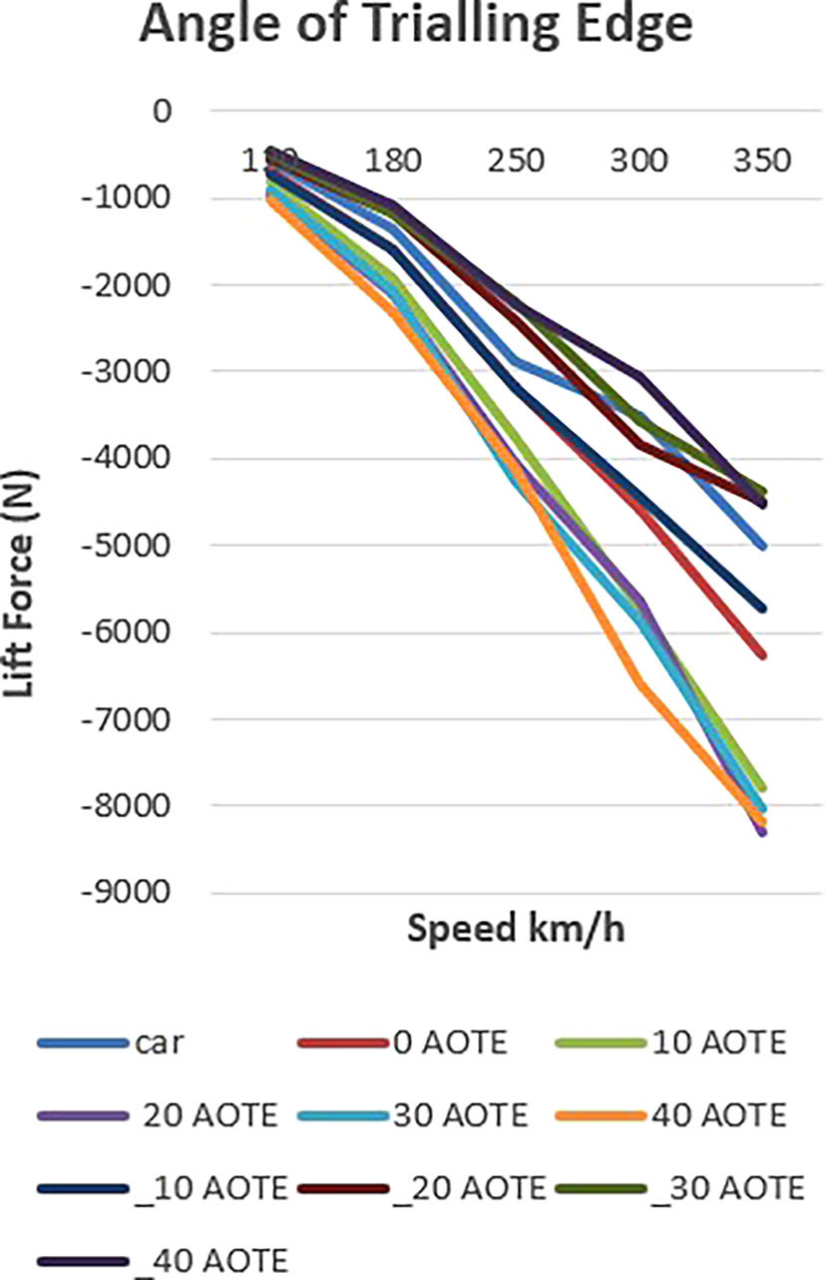
Variation of lift force with different AOTE across speed range.

The relationship between AOTE and lift force across multiple vehicle speeds is shown in Figure 16 and Table 4. Results indicate that increasing AOTE (up to 30°) consistently enhances negative lift (downforce), particularly at high speeds. For instance, at 350 km/h, downforce increases from –5015 N (no spoiler) to –8043 N at 30° AOTE.

**Table 4.** Lift force (FL) and coefficient of lift (CL) at various AOTE values and speeds.

		120 (KM/H)	180 (KM/H)	250 (KM/H)	300 (KM/H)	350 (KM/H)
**Car**	FL (N)	-573.2	-1360	-2886	-3505	-5015
	CL	-0.368	-0.388	-0.427	-0.36	-0.379
**0 AOTE**	FL (N)	-701.4	-1587.1	-3182	-4584	-6264
	CL	-0.437	-0.44	-0.456	-0.457	-0.458
**10 AOTE**	FL (N)	-809.12	-1932	-3746	-5723	-7793
	CL	-0.503	-0.534	-0.537	-0.57	-0.57
**20 AOTE**	FL (N)	-952	-2100	-4060	-5631	-8298
	CL	-0.58	-0.575	-0.578	-0.5553	-0.6
**30 AOTE**	FL (N)	-901	-2086	-4254	-5867	-8043
	CL	-0.56	-0.568	-0.6	-0.575	-0.58
**40 AOTE**	FL (N)	-1016	-2312	-4110	-6609	-8183
	CL	-0.561	-0.62	-0.45	-0.564	-0.58
**-10 AOTE**	FL (N)	-722.22	-1593	-3166	-4449	-5725
	CL	-0.45	-0.44	-0.402	-0.443	-0.419
**-20 AOTE**	FL (N)	-545.5	-1180	-2394	-3840	-4487
	CL	-0.337	-0.324	-0.341	-0.38	-0.326
**-30 AOTE**	FL (N)	-481	-1178	-2197	-3561	-4369
	CL	-0.295	-0.321	-0.31	-0.345	-0.315
**-40 AOTE**	FL (N)	-467	-1070	-2219	-3057	-4530
	CL	-0.294	-0.3185	-0.28	-0.3105	-0.3

**Figure 17.**
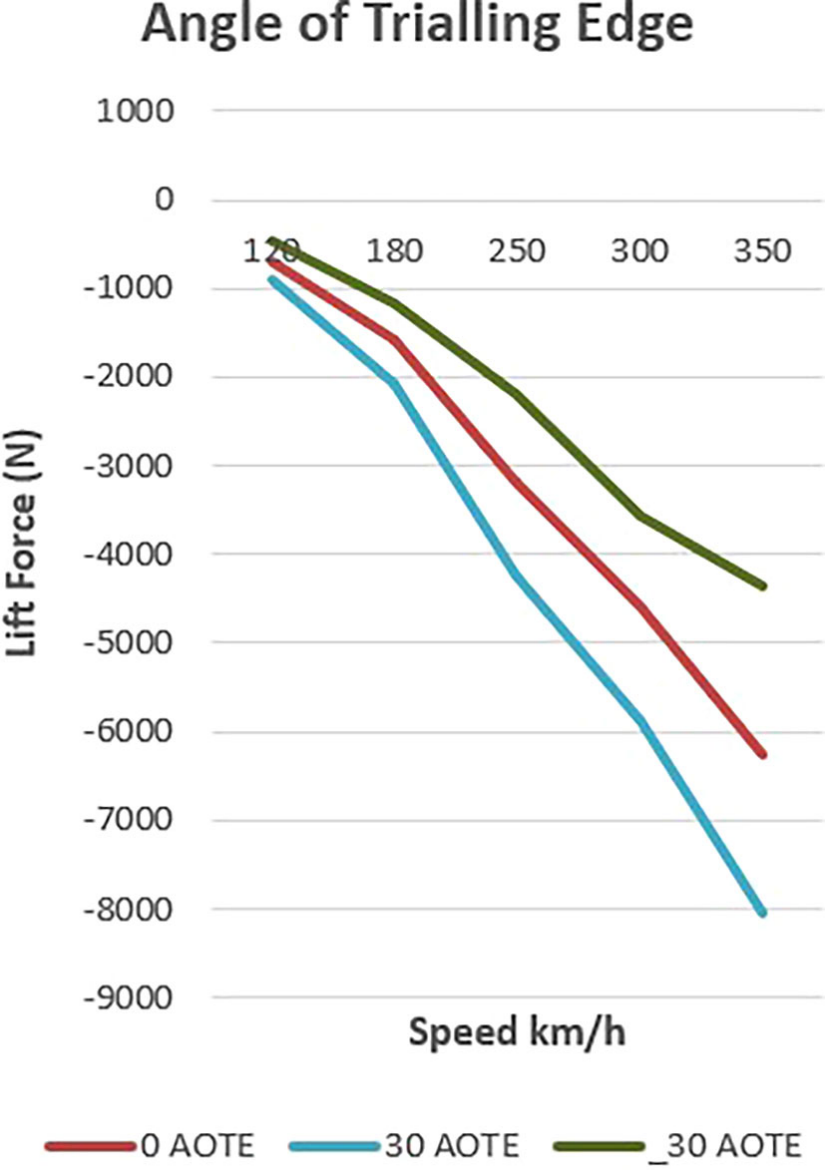
Down force at (-30, 0, 30) AOTE.

The presented Figure 17 delineates the influence of controlled downforce manipulation on vehicle dynamics, specifically focusing on lift and downforce. In the baseline scenario, the vehicle exhibits a progressive increase in downforce with rising velocity, reflecting its inherent design characteristics.

Upon the introduction of a spoiler mechanism and subsequent adjustments to its configuration, a discernible alteration in lift and downforce profiles is observed. This controlled manipulation of aerodynamic forces enables a targeted optimization strategy aimed at augmenting both stability and efficiency metrics.

Lowering the spoiler configuration results in a reduction of downforce, thereby mitigating lift and enhancing overall aerodynamic efficiency. This reduction translates into diminished aerodynamic drag, consequently improving the vehicle’s efficiency profile. Conversely, elevating the spoiler configuration yields an amplification of downforce, concurrently bolstering vehicle stability, particularly during high-speed maneuvers where lateral stability is critical.

Notably, the depiction of lift and downforce values as negative underscores their respective orientations. This negative representation signifies their downward and upward forces, respectively. Thus, even amid downforce reduction, stability remains preserved, mitigating potential risks associated with compromised stability, such as lateral instability or loss of traction.

In summary, the chart elucidates the efficacy of controlled downforce manipulation in optimizing vehicle dynamics. Through strategic adjustments to spoiler configuration, this study presents a scientifically grounded approach to concurrently enhancing stability and efficiency without compromising safety or performance criteria in vehicular engineering.

**Figure 18.**
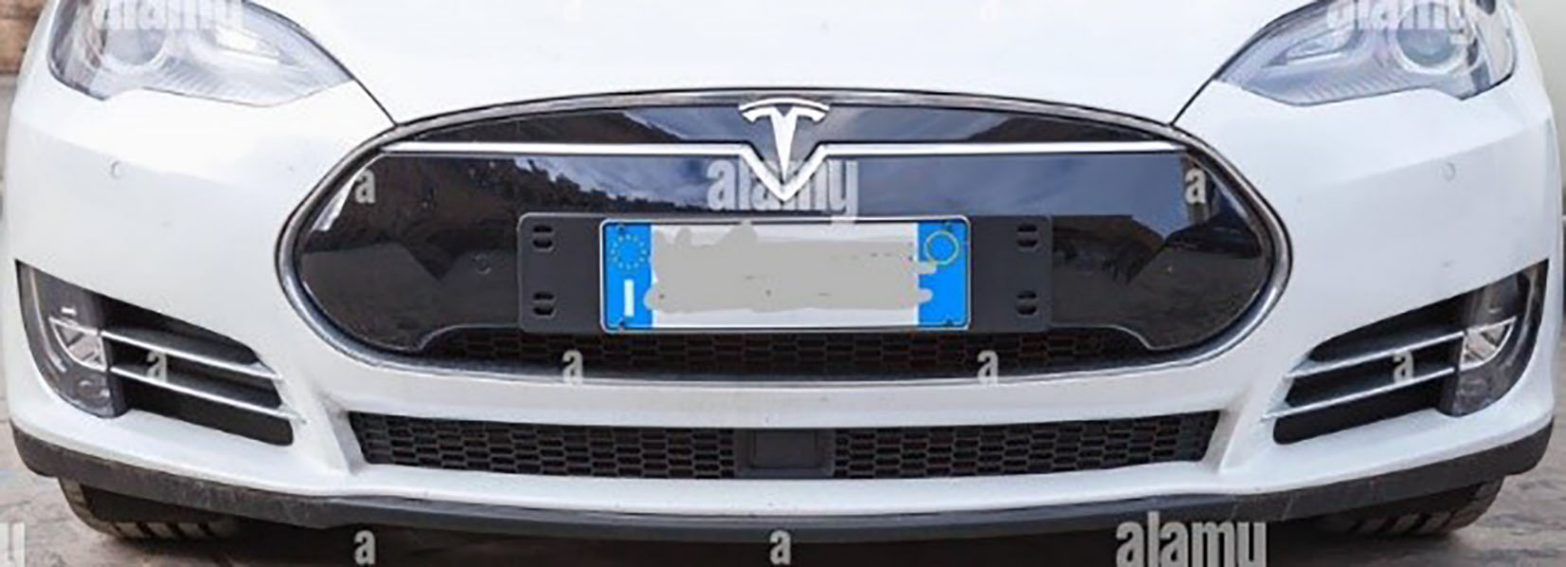
Real-world Tesla Model S (2015) front bumper with drag-reducing features.

**Figure 19.**
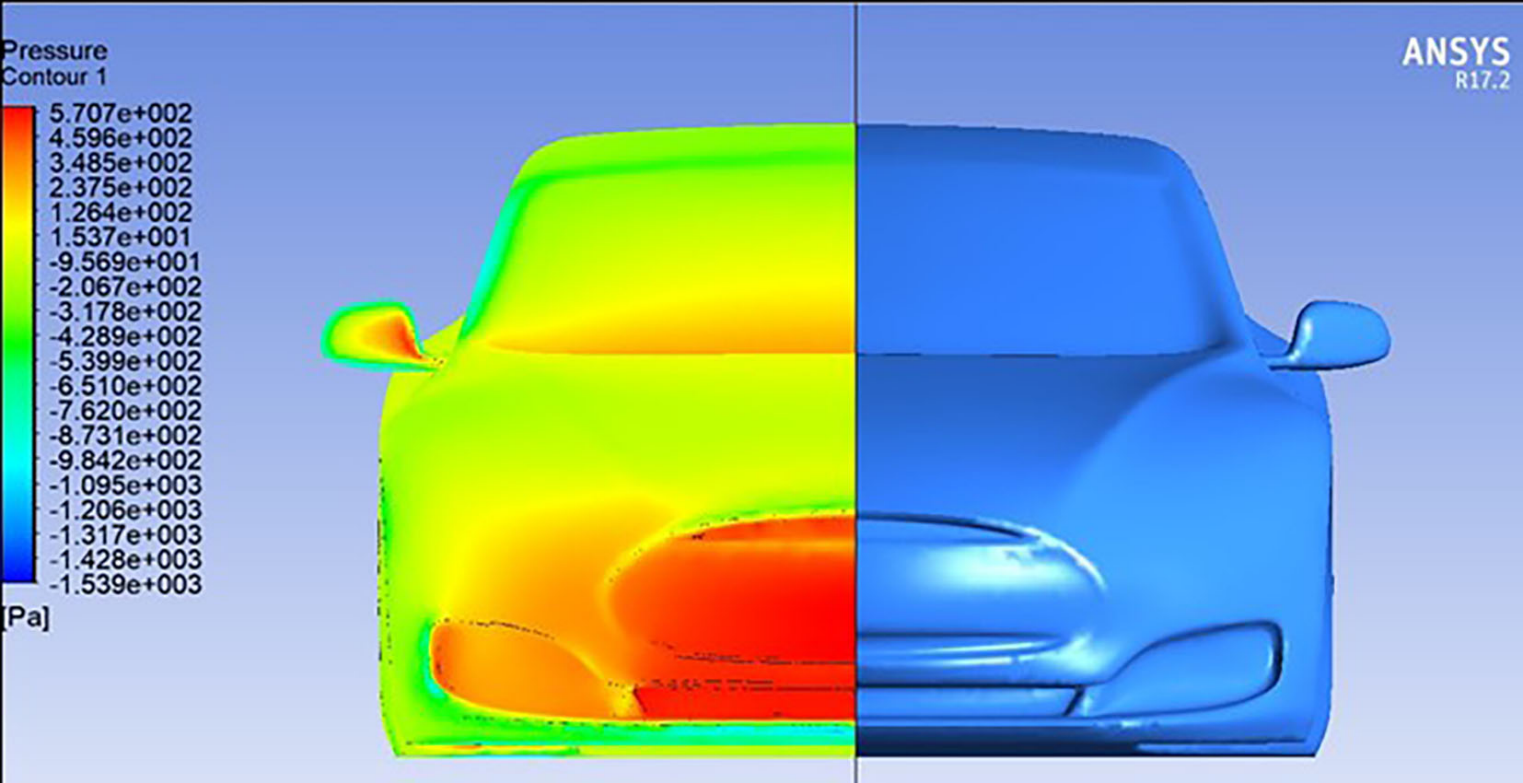
Pressure contour showing airflow interaction with the simplified bumper design.

 Drag Force

The simulated drag coefficient was 0.256, representing a 6% deviation from Tesla’s reported value of 0.24 ^9^.This discrepancy can be attributed to two primary factors:

1. **Mesh Refinement**: The computational mesh used in the simulation, while adequate for initial analysis, could be further refined to enhance accuracy. A higher-resolution mesh would likely produce aerodynamic data more closely aligned with real-world measurements.
2. **Front Bumper Design**: The actual Tesla Model S incorporates perforations in the front bumper, as depicted in Figure 18, Figure 19 which illustrate design features intended to reduce drag. These features were not replicated in the simulation, resulting in a higher simulated drag coefficient.

**Figure 20.**
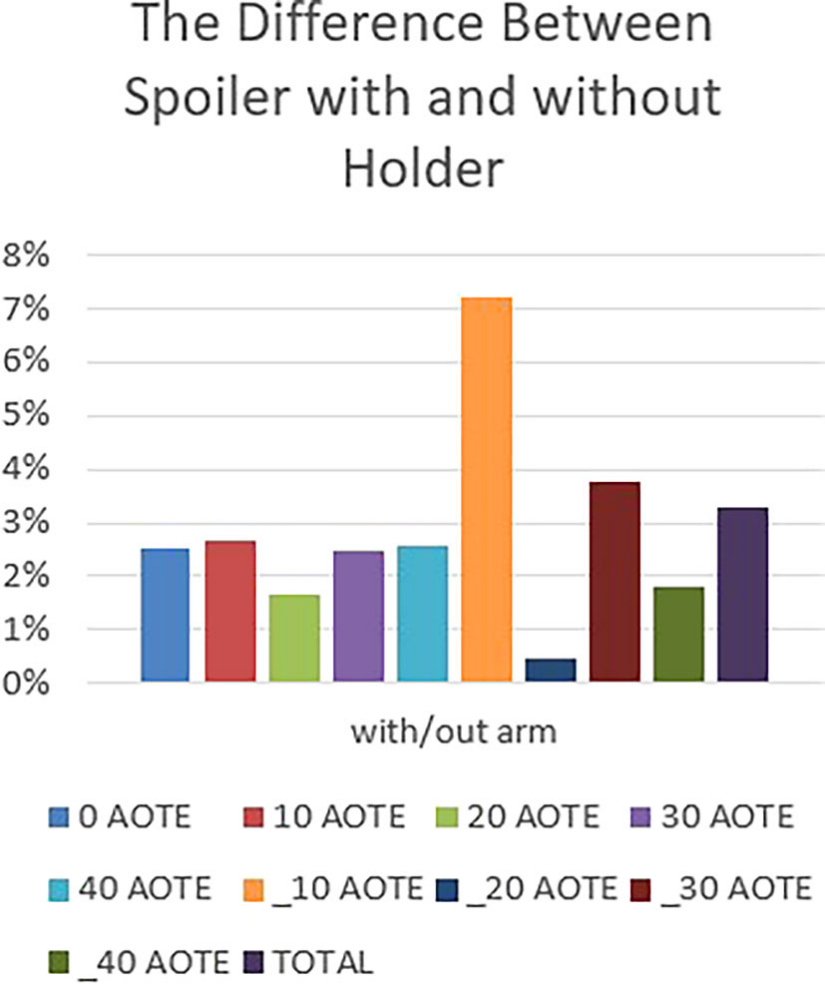
The difference between spoiler with and without a holder.

To address this discrepancy, future simulations should focus on improving mesh resolution and incorporating detailed design elements, such as the front bumper perforations. These adjustments would enable the simulation results to more accurately reflect the aerodynamic performance reported by Tesla. The drag coefficients for various spoiler configurations are presented in Table 5.

**Table 5.** Drag coefficients (CD) for base car and various AOTE spoiler configurations.

	CD
Tesla car	0.24
Simulated car	0.256
Car with spoiler at -30 AOTE	0.278
Car with spoiler at 0 AOTE	0.2823
Car with spoiler at 30 AOTE	0.306

The simulations were conducted in compliance with Tesla’s testing guidelines, which specify a test speed of **70 mph** for determining aerodynamic coefficients. This ensures that the simulation conditions are consistent with real-world testing protocols, providing a reliable basis for comparison.

**Spoiler with and without holder effect**

The placement of a rear spoiler on a vehicle, whether with or without stands (i.e., feet or holders), ^10, 11^ can affect its aerodynamic performance. This study investigates this phenomenon by analyzing lift force data obtained from spoilers with and without stands. Comparing the results highlights notable differences in aerodynamic behavior based on stand placement. This underscores the importance of stand configuration in rear spoiler design and its implications for optimizing vehicle aerodynamics.

The graph in Figure 20 indicates a clear difference between the results obtained for the spoiler with or without a holder (stand, feet, etc.). This variation highlights the aerodynamic influence of the holder’s design, shape, and placement. It emphasizes the importance of carefully considering these factors in holder design to optimize aerodynamic performance and ensure effective integration with the vehicle’s overall aerodynamic profile.

## Conclusion

This project, which explored the use of airfoils with movable trailing edges as spoilers, provided valuable insights into automotive dynamics. The key findings are as follows:

(1) **Dynamic downforce control**: Movable trailing edges allow real-time downforce adjustments, enhancing vehicle stability across driving conditions.
(2) **Enhanced stability and handling**: By dynamically increasing or decreasing downforce, the vehicle maintains optimal grip and balance, especially during high-speed driving and cornering. This results in improved safety, better handling, and increased driver confidence, reducing the risk of skidding and loss of control.
(3) **Improved acceleration**: The ability to reduce downforce when it is not needed, such as during straight-line acceleration, minimizes the negative impact of excessive downforce on speed. This leads to improved acceleration performance, allowing the vehicle to achieve faster and more efficient speed increases without compromising stability.
(4) **Feasibility of integration**: The project has demonstrated that integrating this advanced aerodynamic feature into existing vehicle systems is feasible without extensive modifications. This practical aspect facilitates easier adoption and implementation within the automotive industry.
(5) **Future research and development**: Further research is recommended to optimize the control algorithms and fully explore the benefits of this technology under a wider range of conditions. Continued development will focus on maximizing performance gains, ensuring reliability, and assessing long-term durability.

In conclusion, the application of airfoils as spoilers with movable trailing edges represents a significant advancement in vehicle performance enhancement.

This innovative approach allows for precise downforce modulation, leading to improved stability, handling, and acceleration. The findings provide a solid foundation for future developments, highlighting the potential for widespread application of this technology to enhance vehicle performance, efficiency, and safety.

### Note

Some of the simulated data has been attached to a public database that you can view. ^12^

### Ethics and consent

Ethical approval and consent were not required for this study, as it did not involve human participants, animal subjects, or sensitive data. The research focused solely on computational simulations and analysis of aerodynamic performance using publicly available data and software tools.

## Data Availability

The data can be accessed at: https://doi.org/10.5281/zenodo.14602400. ^12^ Data are available under the terms of the Creative Commons Zero “No rights reserved” data waiver (CC0 1.0 Public domain dedication).

## References

[1] Smith A Johnson B : Aerodynamic design of automotive spoilers. *Journal of Vehicle Engineering.* 2018;25(3):123–140.

[2] Patel S Garcia M : Optimizing automotive spoiler design for improved aerodynamic performance. *International Journal of Vehicle Design.* 2021;35(2):145–162.

[3] Thompson C : Effects of spoiler configurations on speed and handling. *International Journal of Automotive Performance.* 2019;12(2):78–92.

[4] Chen X : Stability enhancement and rollover prevention with automotive spoilers. *Journal of Vehicle Safety.* 2017;22(4):321–340.

[5] Green R : Environmental considerations in automotive spoiler design. *Journal of Sustainable Transportation.* 2022;40(1):56–75.

[6] Lee H Kim J : Design integration of spoilers in vehicles for improved efficiency. *International Journal of Automotive Engineering.* 2019;29(4):321–336.

[7] Menter FR : Zonal two equation k-ω turbulence models for aerodynamic flows. *AIAA Paper.* 1993;93–2906.

[8] Menter FR : Two-equation eddy-viscosity turbulence models for engineering applications. *AIAA J.* 1994;32(8):1598–1605.

[9] Tesla: *Five slippery cars enter a wind tunnel.* Tesla;2024, August 16. Reference Source

[10] Youssef MI : Using CFD analysis to investigate the appropriate height of the rear spoiler on a car. *Zenodo.* 2022, August. 10.5281/zenodo.7036018

[11] Kozak J : Car design as a new conceptual solution and CFD analysis in purpose of improving aerodynamics. *Journal of Automotive Engineering.* 2014;15(3):45–60. Reference Source

[12] Karaki A : Enhancing vehicle performance through the application of airfoils as spoilers with movable trailing edge (Ansys.Products.17.2.Win64). *Zenodo.* 2025. 10.5281/zenodo.14602400 PMC12171717 40529636

