# Supplementary material for: Enhancing vehicle performance through the application of airfoils as spoilers with movable trailing edge
Source: F1000Res. 2025 Jun 9;14:469. Originally published 2025 Apr 28. [Version 2] doi: 10.12688/f1000research.160307.2 (PMC12171717; doi:10.12688/f1000research.160307.2)
Supplement: Peer review history

# Reviewer report: Reviewer response for version 2

**DOI:** 10.5256/f1000research.183367.r390484

**Published:** 2025-06-16

**Authors:** Mustafa S. Abood

**Affiliations:** 1 University of Baghdad, Baghdad, Baghdad Governorate, Iraq

The revised article addresses my previous concerns effectively. The abstract, methodology, and literature review have been improved, and the language is clearer. Minor issues like figure resolution remain, but they do not affect the validity of the work. I have no further major comments and consider the article suitable for approval.

Is the work clearly and accurately presented and does it cite the current literature?

Partly

If applicable, is the statistical analysis and its interpretation appropriate?

Not applicable

Are all the source data underlying the results available to ensure full reproducibility?

Yes

Is the study design appropriate and is the work technically sound?

Yes

Are the conclusions drawn adequately supported by the results?

Yes

Are sufficient details of methods and analysis provided to allow replication by others?

Yes

Reviewer Expertise:

My areas of expertise include aerodynamics, computational fluid dynamics (CFD), and heat transfer. I have extensive experience with engineering simulation software, and my research focuses on thermal-fluid sciences, including both theoretical and applied aspects of fluid mechanics and thermodynamics.

I confirm that I have read this submission and believe that I have an appropriate level of expertise to confirm that it is of an acceptable scientific standard.

# Reviewer report: Reviewer response for version 1

**DOI:** 10.5256/f1000research.176184.r381823

**Published:** 2025-05-28

**Authors:** Mustafa S. Abood

**Affiliations:** 1 University of Baghdad, Baghdad, Baghdad Governorate, Iraq

**Peer Review Report**

**Article Title:**

*Enhancing vehicle performance through the application of airfoils as spoilers with movable trailing edge*

**Summary**

 This research article explores the aerodynamic impact of adjustable spoilers with movable trailing edges on a vehicle’s performance using CFD simulations in ANSYS Fluent. A Tesla-like model was designed and tested under various conditions to assess lift, drag, and downforce metrics. The results suggest meaningful gains in stability and handling, depending on the spoiler configuration.

 The topic is relevant and the work is technically sound, with a strong methodology and well-executed simulation workflow. The authors demonstrate a clear understanding of aerodynamic principles and CFD simulation practices. However, several presentation, grammatical, and clarity issues reduce the manuscript’s overall impact and readability.

**Assessment**

**1. Is the work clearly and accurately presented, and does it cite the current literature?**

**Partially.**

- The article is generally well-structured, but contains **numerous grammatical issues, awkward phrasing, and inconsistent figure references**.
- The literature review is **adequate**, though it could benefit from **stronger integration with the study’s goals** (e.g., what gaps this specific paper fills).
- Figures and tables are useful but **would benefit from clearer captions** and smoother integration in the discussion.

**2. Is the study design appropriate and does the work have academic merit?**

**Yes.**

- The methodology using **CFD with SST k-ω turbulence modeling** is sound and appropriate.
- Simulation conditions and meshing are reasonably justified.
- The **comparison of multiple AOTE values** adds useful depth to the study.

**3. Are sufficient details of methods and analysis provided to allow replication by others?**

**Yes, mostly.**

- The authors provide good detail on the simulation setup, boundary conditions, and spoiler geometry.
- Some **minor clarifications** are needed, e.g., regarding how angle increments were chosen and how grid independence was tested.

**4. If applicable, is the statistical analysis and its interpretation appropriate?**

**Not applicable.**

- The paper relies on **deterministic CFD simulation results** rather than statistical hypothesis testing. However, comparative trends are clearly discussed.

**5. Are all the source data underlying the results available to ensure full reproducibility?**

**Yes.**

- The dataset is available via Zenodo, which supports reproducibility.

**6. Are the conclusions drawn adequately supported by the results?**

**Yes.**

- The conclusions are aligned with the findings.
- The authors are **appropriately cautious**, acknowledge limitations, and suggest reasonable directions for future work.

**Constructive Criticisms & Suggestions**

**Major Suggestions:**

1. **Language Revision:**
  - The manuscript needs **professional editing or proofreading** to improve clarity and grammar.
  - Phrasing such as “determiningaerodynamic” (no space) appears throughout.
2. **Figures and Captions:**
  - Improve **figure referencing and explanation** in the body text. Ensure every figure (e.g., Figure 11–18) is described clearly.
  - Some images lack scale or axis labels—consider adding where applicable.
3. **Abstract Improvements:**
  - The abstract is informative but could better reflect the **key numerical findings** (e.g., drag reduction %, stability improvements).
4. **Literature Integration:**
  - While literature is cited, the authors should **better contrast their findings** with existing results to highlight novelty.

**Minor Suggestions:**

- Clarify the **reason for selecting the NACA0012 airfoil** beyond its symmetry; include references if it's a standard in spoiler design.
- Specify the **mesh resolution** or number of elements used.
- Include a **brief explanation of computational cost** or runtime to guide other researchers.

**Recommendation**

**Approval Status:** *Approved with Reservations*

 The study has academic merit, a valid methodology, and useful findings. However, the manuscript needs language polishing and better figure integration to meet F1000Research’s indexing standards.

Is the work clearly and accurately presented and does it cite the current literature?

Partly

If applicable, is the statistical analysis and its interpretation appropriate?

Not applicable

Are all the source data underlying the results available to ensure full reproducibility?

Yes

Is the study design appropriate and is the work technically sound?

Yes

Are the conclusions drawn adequately supported by the results?

Yes

Are sufficient details of methods and analysis provided to allow replication by others?

Yes

Reviewer Expertise:

My areas of expertise include aerodynamics, computational fluid dynamics (CFD), and heat transfer. I have extensive experience with engineering simulation software, and my research focuses on thermal-fluid sciences, including both theoretical and applied aspects of fluid mechanics and thermodynamics.

I confirm that I have read this submission and believe that I have an appropriate level of expertise to confirm that it is of an acceptable scientific standard, however I have significant reservations, as outlined above.

# Reviewer report: Reviewer response for version 1

**DOI:** 10.5256/f1000research.176184.r384250

**Published:** 2025-05-28

**Authors:** Aniello Riccio

**Affiliations:** 1 University of Campania “Luigi Vanvitelli”, via Roma, Aversa, Italy

Review on Enhancing vehicle performance through the application of airfoils as spoilers with movable trailing edge.

 The proposed work presents a numerical study conducted by means of CFD simulations with the aim of analysing and optimising the aerodynamic effect of moving trailing edge spoilers applied to a simplified vehicle model (Tesla).

 The topic is current and relevant to the automotive industry. The study is well structured, and the technical content is valid. However, there are a few points that need clarification or further investigation. The following comments are suggested:

1. The state of the art is poor. Please, improve it. The following paper are given as suggestion:

- *Riccio A., Sellitto A., Battaglia M. Morphing Spoiler for Adaptive Aerodynamics by Shape Memory Alloys (2024) Actuators, 13 (9), art. no. 330*

*-Battaglia, M.; Sellitto, A.; Giamundo, A.; Visone, M.; Riccio, A. Shape Memory Alloys Applied to Automotive Adaptive Aerodynamics. Materials 2023, 16, 4832.*

1. According to the state of the art, what are the innovative aspects compared to existing studies on active or adaptive spoilers? Please, put more emphasis on this aspect.
2. According to the CFD simulation, did authors  perform a sensitivity analysis on mesh or turbulent parameters to confirm the robustness of the results? Please, clarify.
3. Please, be sure that all the terms of the equations have been defined in the text.
4. How were the speed range chosen? Are the values 120-350 km/h based on realistic conditions?
5. Paragraphs and sub-paragraphs should be better organised.
6. Please, improve the quality of figures (e.g. figures 18,19)

Decision: Minor revision.

Is the work clearly and accurately presented and does it cite the current literature?

Yes

If applicable, is the statistical analysis and its interpretation appropriate?

Yes

Are all the source data underlying the results available to ensure full reproducibility?

Yes

Is the study design appropriate and is the work technically sound?

Yes

Are the conclusions drawn adequately supported by the results?

Yes

Are sufficient details of methods and analysis provided to allow replication by others?

Yes

Reviewer Expertise:

aerospace, computational mechanics, additive manufacturing, adptive aerodynamics for automotive application

I confirm that I have read this submission and believe that I have an appropriate level of expertise to confirm that it is of an acceptable scientific standard.
